# Parents’ and informal caregivers’ experiences of accessing childhood vaccination services within the United Kingdom: a systematic scoping review of empirical evidence

**DOI:** 10.1186/s12889-024-20981-0

**Published:** 2024-12-18

**Authors:** Georgia Chisnall, Samar Hersh-Toubia, Sandra Mounier-Jack, Louise Letley, Tracey Chantler

**Affiliations:** 1 London School of Hygiene and Tropical Medicine, Keppel Street, London, WC1E 7HT UK; 2 AVA (Against Violence & Abuse), The Foundry, 17 Oval Way, London, SE11 5RR UK; 3 Immunisation and Vaccine Preventable Diseases Division, Health Security Agency, 10 South Colonnade, Canary Wharf, London, E14 4PU UK

**Keywords:** Childhood vaccination, Immunisation, Parents, Health services, Accessibility

## Abstract

**Background:**

Despite repeated calls to action and considerable attention, childhood vaccination uptake has declined for a thirteenth consecutive year in the United Kingdom (UK). Increasingly, stakeholders are advocating for research which goes beyond vaccine hesitancy and explores service accessibility in greater depth. This scoping review aims to identify and critically assess how accessibility is being conceptualised and investigated with a view to informing future research. Research, that in turn, will dictate the interventions pursued to improve vaccination coverage.

**Methods:**

A detailed search strategy was implemented across seven databases to identify research exploring parents’ experiences of accessing childhood vaccination services within the UK. The analysis explored the studies in relation to their conceptualisation of access, methodology, reported results, and recommendations for research or practice using a combination of descriptive qualitative content analysis, typologies, and frequency counts. Methods and reporting adhered to the ‘JBI Manual for Evidence Synthesis’ and the ‘Preferred Reporting Items for Systematic Reviews and Meta-Analyses for Scoping Reviews’.

**Results:**

Forty-five studies were included in the analysis. Studies claimed to consider only attitudinal constructs (4%) or did not discuss access at all (33%) despite findings, in part, including access related issues. Remaining studies used the term access in passing or ambiguously (24%), distinguished between attitudes and access in-text (27%), and a minority of studies utilised a theoretical framework which acknowledged accessibility (13%). The focus on access to information (92% of studies) was disproportionately large compared to other domains of accessibility such as availability (11%), affordability (13%), and proximity (16%). Of the seven identified intervention studies, five were centred on information provision.

**Conclusion:**

Accessibility is poorly conceptualised within most of the research conducted on childhood immunisation uptake within the UK. This, in part, is because exploring accessibility was not an explicit objective of many of the studies included in the review. It is vital that the accessibility of childhood vaccination services is given greater priority and appropriately defined in empirical research. Otherwise, researchers run the risk of limiting the scope of their findings based on their own conceptual ideas regarding the drivers of poor uptake rather than the lived reality of parents.

**Supplementary Information:**

The online version contains supplementary material available at 10.1186/s12889-024-20981-0.

## Introduction

Immunisation is a cornerstone of public health, representing both a highly successful and cost effective prevention programme [1]. The national immunisation programme within the United Kingdom (UK) is voluntary and provides vaccinations free of charge, aiming to give equal opportunity for protection against vaccine preventable disease [1, 2]. Routine vaccination forms a key part of the Healthy Child Programme and is said to lie ‘*at the heart of universal services for children and families*’ [1]. Vaccination has resulted in significant reductions in numerous communicable diseases including diphtheria, rubella, Haemophilus influenza type b, meningococcal group C disease, and polio [2]. Beyond individual and societal protection in terms of reduced morbidity and mortality, outcomes include the reduction of hospital admissions and reduced use of antimicrobials [1].

**Fig. 1:**
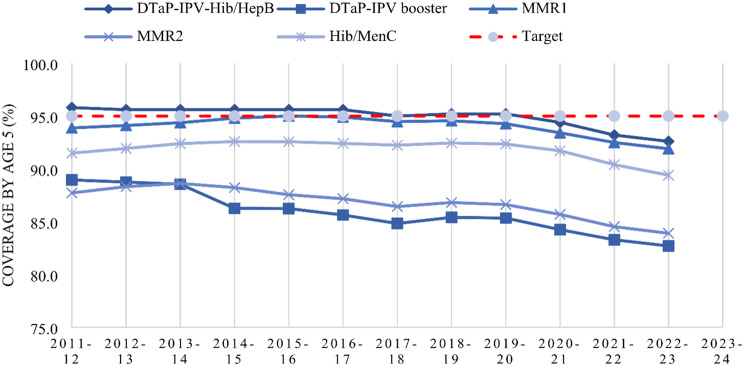
Percentage of children vaccinated by their fifth birthday. Note, data taken from NHS Digital [3]. The DTaP/IPV/Hib (5-in-1) vaccine was replaced by the DTaP/IPV/Hib/HepB (6-in-1) vaccine in August 2017, thus 2013–2023 reflects coverage for the 5-in-1 vaccine

While the ‘*overwhelming majority*’ of parents in England automatically vaccinate their children and acceptance ‘*is at the highest level*’, challenges with vaccine uptake remain [2]. In fact, childhood vaccination coverage has decreased for a thirteenth consecutive year in the UK (see Fig. 1) [3]. The British Medical Association has voiced concern about this declining trend and called for long-term investment to improve the uptake of vaccination services in line with the 95% target set by the World Health Organisation [4]. This threshold of vaccination coverage is necessary to control disease spread and pursue disease elimination [4]. Beyond vaccine uptake, delayed vaccination is also jeopardising the immunisation programme leaving children and communities vulnerable during the period of non-vaccination [1, 5]. For instance, coverage of the first dose of MMR drops from 91.9 to 88.9% when captured at age two compared to age five, despite being due at age one [3].

Despite the pervasive perception, particularly propagated by media, that ‘*anti-vaxxers*’ are to blame for sub-optimal vaccination coverage, in reality *‘…with a few small exceptions*,* it is hard to find a powerful anti-vax group or movement today that has a substantial impact’* [6]. Contrary to common belief, for the vast majority of parents this delay is associated with challenges in accessing services in a timely manner rather than ‘*vaccine hesitancy*’ per se [2]: *‘…most under-immunisation in the UK arises out of difficulties with access to vaccination services for parents…*’ [7]. By vaccine hesitancy we mean those who are conflicted about, or opposed to, getting vaccinated and thus decide to refuse or delay vaccination despite services being available to them [8]. Vaccine hesitancy is complex and context specific, but can be driven by low trust in the effectiveness, safety, or need for vaccination [8].

A national survey (*n* = 1792) found that of the 10% of parents who did not take their children for vaccination when due, only 2% were due to vaccine refusal [2]. As Dowden puts it, *‘Sometimes it pays not to make assumptions about what is driving pockets of poor vaccination*,* but to focus on helping stretched parents who may simply be having a hard time accessing services’* [6]. Countries with high levels of vaccine confidence and demand can still experience suboptimal childhood vaccination coverage, highlighting the ‘*often overlooked*’ but important issue of accessibility [9]. Stakeholders in childhood vaccination have stated the need to be more inclusive of non-attitudinal factors in improving the utilisation of vaccination services [10].

Socio-economic challenges faced by parents such as transport, childcare and competing household work may impact vaccine service accessibility [6, 9, 11, 12]. Alternatively, features of the vaccination service or delivery logistics such as waiting times and organisational procedures (e.g., rigid booking systems, lack of recall and reminders, or poor record keeping) may have a bearing [6, 9, 11, 12]. Similarly, interactions with frontline healthcare workers including information provision and communication (e.g., information volume, positive bias, format accessibility, or timeliness) can hinder accessibility [9, 11, 12]. Vulnerable populations (e.g., children in the welfare system, migrants, travellers, or military workers) report specific access barriers such as impermanent residence and health system exclusion or poverty. This can diminish trust in government services which reduces the extent to which individuals feel comfortable and confident accessing healthcare programmes [6, 9]. This experience is driven not only by fellow service users but also by the societally held beliefs and values of healthcare workers [6, 9].

### Why is it important to do this synthesis?

Historically, the literature on parental vaccination behaviours was heavily focused on vaccine attitudes. This is reflected in the use of models to understand vaccine uptake which exclusively considers health behaviour from a decision-making perspective, such as the Health Belief Model or the Theory of Planned Behaviour [9]. There has been a growth in the consideration given to drivers of vaccine uptake and not labelling sub-optimal coverage automatically as vaccine hesitancy. This has resulted in the publication of multiple systematic reviews [9, 11, 13], including a number of evidence syntheses commissioned as part of the guideline development process on increasing uptake of vaccinations within the UK by the National Institute for Health and Care Excellence (NICE) [14–17].

While systematic reviews are crucial in informing specific, trustworthy guidelines or interventions based on the current available evidence, these forms of review are not designed to answer broader more exploratory questions regarding the research field itself [18, 19]. For instance, the types of evidence and concepts that underpin a research field, working definitions, or conceptual boundaries [19, 20]. Unlike systematic reviews, the aim of a scoping review is not to report singular results which stem from a highly specific quality assessed synthesis, but to map the field. Hence, scoping reviews has been referred to as gaining an understanding of the “lay of the land” [21]. Scoping reviews are essential in providing direction for future research, by ensuring that devised projects are appropriately built upon the pre-existing landscape [21]. In other words, breadth is pursued over depth to guide or re-direct subsequent, more focused, lines of research.

Hence, this scoping review aimed to identify and critically assess how accessibility is being conceptualised and investigated with a view to informing future research. Research, that in turn, will dictate the interventions pursued to improve vaccination coverage. To the authors’ knowledge, no such scoping review exists on this topic as per the outcome of searches conducted in Cochrane Library, Campbell Collaboration, PROSPERO and EPPI-Centre publications.

### Objectives and research questions

The objectives and research questions of the review are outlined in Table 1.

**Table 1:** Review objectives and research questions

Objectives	Research questions
1. To map key concepts/definitions considered by the existing literature in relation to the accessibility of childhood vaccination services in the UK.	a. How is accessibility conceptualised/defined within UK literature? b. What are service users’ views and experiences of accessing childhood vaccination services in the UK? c. What are the key factors that impact the accessibility and user-friendliness of childhood vaccination services within the UK? d. What recommendations have been made to improve the experience and/or accessibility of childhood vaccination services within the UK.
2. To report on the types of methods/evidence which have been used to explore the accessibility of childhood immunisation services in the UK.	a. What theoretical constructs have been used to guide UK-based research in this area? b. What methods have been used to explore experiences/accessibility of childhood vaccination services within the UK? c. Which populations/groups have been consulted as service users of childhood vaccination services within the UK?
3. To identify gaps in the research/knowledge base on the accessibility of childhood services within the UK.	a. What calls to action for research have been made within the literature? b. What gaps have emerged as a result of answering the previous research questions (in terms of concepts and/or methods)?

## Methods

Methods were developed using the JBI Manual for Evidence Synthesis [18]. Reporting adheres to the Preferred Reporting Items for Systematic Reviews and Meta-Analyses for Scoping Reviews checklist (PRISMA-ScR) [22]. Supporting guidance was provided by Lockwood, Dos Santos [23]. The review protocol is available at 10.31219/osf.io/dzbrw.

## Eligibility criteria

While systematic reviews require a narrow mnemonic such as ‘population, intervention, comparator, outcome’ (PICO), scoping reviews tend to use the less restrictive criterion of ‘population, concept, context’ (PCC). This facilitates a boarder exploration of the research area as per the review’s objectives [18, 23]. We included any primary empirical literature which met the PCC criterion detailed below, whether this be qualitative, quantitative, or mixed methods in design.

Non-empirical articles were excluded (e.g., opinion articles, editorials, news publications, or conference abstracts). Grey literature often focuses on disseminating conclusions rather than the methodological process through which they were reached [24]. Given the objectives of the review to explore how accessibility is conceptualised within empirical research, how it is investigated, and what areas for future research have been suggested (i.e., being highly dependant on extracting data from the methods section) this was not need deemed appropriate. Nonetheless, the limitations of excluding grey literature are considered in the discussion.

No limitations were placed on the year of publication. While the vaccine programme has undergone several changes over the years it was felt that any factors which affect service accessibility may still have relevance today. Furthermore, as a scoping review capturing the research field in full was preferable. After piloting only one factor was added to the original eligibility criteria: simple coverage or epidemiological data depicting vaccine uptake per characteristic (e.g., maternal mental health records) did not meet the threshold for satisfying the concept criterion of accessibility or experience of service use – unless this was in relation to an accessibility or service satisfaction related intervention. However, these studies were tagged in Rayyan and are briefly discussed in the study screening section.

### Population

Parents or informal caregivers with real-life experience of accessing vaccination services are defined as; anyone who is directly involved in childcare, decisions on vaccination, or facilitation of vaccination. Papers that only reported on the topic of interest from the perspective of other stakeholders in childhood vaccination such as healthcare workers, policymakers, or programme administrators were excluded.

### Concept

We focused on factors influencing the views and experiences of parents and informal caregivers accessing childhood immunisation services within the UK. Here we sought to explore non-attitudinal factors such as parental or caregiver socioeconomic realities, or challenges associated with the inherent features of vaccination services. We did not include studies which: exclusively focused on attitudinal factors (i.e., vaccine hesitancy) such as distrust or fear; or explored the topic of interest from a hypothetical, intention to vaccinate, perspective; this included predicative modelling papers. Hypothetical (or intention) studies were excluded because these could not capture the lived reality of accessing childhood vaccination services. In asking parents whether they planned to vaccinate their child the onus was on the decision-making of the parent, rather than the structural barriers to vaccination which they had not yet encountered.

We used qualitative content analysis to distinguish whether papers considered accessibility factors, in addition to attitudinal factors, as reported by Kaufman, Tuckerman [9]. In instances of uncertainty during the title and abstract screening articles progressed to full-text screening to avoid missing relevant articles; this would have included articles that were published prior to the popularisation of the term ‘vaccine hesitancy’. Instances where articles were excluded during the title and abstract screening included papers that reported exclusively focusing on ‘vaccine hesitancy’ or ‘anti-vax’.

For the purposes of this review, childhood vaccination includes vaccines recommended in the NHS routine and selective childhood immunisation programme [25]. We did not consider those vaccines delivered in schools (e.g., 12 years and above) as school delivery models side-step the interface between primary health care and parents. We included studies that were in relation to any number or selection of the vaccines listed in Tables 2 and 3, or in relation to the ‘childhood immunisation programme’ as a whole within the UK. Studies on ‘catch-up campaigns’ were also excluded as these do not form part of the routine immunisation programme.

**Table 2:** Routine childhood immunisations UK– 2020

Age	Disease protected against	Vaccine
8 weeks	Diphtheria, tetanus, pertussis (whooping cough), polio, Haemophilus influenzae type b (Hib) and hepatitis B	DTaP/IPV/Hib/HepB
	Meningococcal group B (MenB)	MenB
	Rotavirus gastroenteritis	Rotavirus
12 weeks	Diphtheria, tetanus, pertussis, polio, Hib and hepatitis B	DTaP/IPV/Hib/HepB
	Pneumococcal (13 serotypes)	Pneumococcal conjugate vaccine (PCV)
	Rotavirus	Rotavirus
16 weeks	Diphtheria, tetanus, pertussis, polio, Hib and hepatitis B	DTaP/IPV/Hib/HepB
	MenB	MenB
1 year	Hib and MenC	Hib/MenC
	Pneumococcal Measles, mumps and rubella (German measles)	PCV booster MMR
	MenB	MenB booster
Eligible paediatric age group	Influenza	Live attenuated influenza vaccine LAIV^1^
3 years 4 months	Diphtheria, tetanus, pertussis and polio	dTaP/IPV
	Measles, mumps and rubella	MMR

Adapted from [25]

1. If LAIV (live attenuated influenza vaccine) is contraindicated and the child is in a clinical risk group, use inactivated flu vaccine

**Table 3:** Selective childhood immunisations UK– 2020

Age	Target group	Disease protected against	Vaccine
At birth, four weeks and 12 months old^1^	Babies born to hepatitis B infected mothers	Hepatitis B	Hepatitis B (Engerix B/HBvaxPRO)
At birth	Infants in areas of the country with TB incidence > = 40/100,000	Tuberculosis	BCG
At birth	Infants with a parent or grandparent born in a high incidence country	Tuberculosis	BCG
From 6 months to 17 years of age	At risk children	Influenza	LAIV or inactivated flu vaccine if contraindicated to LAIV or under 2 years of age

Adapted from [25]

1. In addition hexavalent vaccine is given at 8, 12 and 16 weeks

### Context

Although there may be some commonalities, vaccination uptake is context-specific and hence there is value in considering vaccination on a country by country basis [9, 12]. This is due to differences in the operational delivery of vaccination programmes between countries, but also the varied contextual cultural factors that shape service provision and use. For instance, some (typically high-income) countries are said to be guided by neoliberal logic where there is a greater onus on parental responsibility, while others are guided by concepts surrounding social exclusion (typically low-income) from services when it comes to vaccine uptake [11]. Therefore, it was deemed appropriate to exclude studies conducted outside of the UK. We focused on factors that influence the views and experiences of parents and informal caregivers in accessing childhood immunisation services within the UK irrespective of vaccination setting or mode of delivery (e.g., healthcare facilities, outreach sites, mobile health teams).

## Information sources

The following electronic databases were searched for eligible studies. The searches were conducted on 27.01.2022–28.01.2022. GC screened the reference lists of relevant reviews identified through the search.

1. Medline (Ovid).
2. Embase Classic + Embase (Ovid).
3. CINAHL (EBSCO).
4. Web of Science Core Collection (Clarivate Analytics).
5. APA PsychINFO (Ovid).
6. Scopus.
7. Social Policy & Practice.

### Search

The draft search strategy was composed of four concepts: parents; experiences; childhood immunisation; and United Kingdom. The draft search was iteratively built in Embase Classic + Embase (OVID). This involved identifying the search concepts, basic key words, synonyms, appropriate truncations, and relevant subject headings. The ‘United Kingdom’ concept search strategy was developed previously by specialists at the London School of Hygiene and Tropical Medicine. The search strategy was peer reviewed by a librarian prior to implementation.

Minor amendments were made to improve the drafted search strategy within the protocol prior to implementation: ‘vaccina*’ was amended to ‘vaccin*’ to also capture the term ‘vaccine’; and subject headings were added for vaccine names where available (e.g., ‘diphtheria pertussis tetanus vaccine/’). The drafted search strategy was adapted, in terms of syntax and subject headings where appropriate, for each of the selected databases. Each of the search strategies devised for each database can be found in Additional File 1.

The results from the search were imported into EndNote and de-duplicated using the stratified de-duplication strategy provided by Leeds University Library as reported by Falconer [26]. The remaining articles were imported into Rayyan ready for title and abstract screening.

### Selection of sources of evidence

Screening took place in two phases, first title and abstract screening (conducted on Rayyan), followed by full-text screening. Following title and abstract screening, articles identified as potentially relevant to the review were sourced in full and stored on a OneDrive folder. Following initial screening by the lead author, a second reviewer undertook a blind review of 50% of title-abstract screening (*n* = 3472) and of full-text exclusions (*n* = 38) as a reliability measure. The blinded agreement rate was 99% for the first wave of screening and 76% for the second wave of full-text screening. According to Belur, Tompson [27], this represents a ‘near perfect’ and ‘substantial’ degree of coding precision respectively. Any discrepancies in screening were resolved through discussion. Details of all articles which progressed to full text screening are provided in Additional File 2 with reasons provided for excluded articles.

### Data charting process and data items

A data charting form was developed in Excel to extract data from each included study. GC piloted this on three studies prior to implementation. Both quantitative and qualitative data were extracted by GC and entered into the form using narrative synthesis. The items included in the data extraction form were as follows:

- Study details
  - Author(s)
  - Year of publication
  - Aims/purpose of the study
  - Vaccine(s) considered

b. Objective 1
  - Studies definition of accessibility
  - Views/experiences
  - Accessibility factors
  - Recommendations to improve accessibility

c. Objective 2
  - Theoretical constructs
  - Methods
  - Populations/groups studied

d. Objective 3
  - Recommendations for research

For intervention studies, data on the intervention, intervention development, and intervention outcome were also extracted. The data extraction forms can be found in Additional File 3.

### Synthesis and presentation of results

Unlike systematic reviews which aim to present a universal, singular truth scoping reviews are trying to capture and represent the breadth and variation within a field. Hence, scoping reviews synthesise the results using a mapping approach in the form of frequency counts and descriptive qualitative content analysis [18]. Results that were synthesised using descriptive qualitative content analysis are presented through narration in the form of typologies (a classification system according to a general type), while results synthesised using frequency counts are presented using figures or percentages. The prevalence of some typologies were in turn used to generate frequency count data.

Mapping the access factors considered within the literature was synthesised utilising an accessibility framework originally developed by Penchansky and Thomas [28] and further developed by Saurman [29]. This framework posits six distinct, but interconnected, dimensions of access as summarised in Table 4. Note, these methods are applied universally whether a study is qualitative, quantitative, or mixed methods in nature as the aim is to map their conceptualisation of the research field, accessibility factors, theories used, populations consulted, and knowledge gaps.

**Table 4:** Theoretical framework for access

Dimension of access	Brief definition	Expanded definition
*Accessibility* ^*1*^	Location	Proximity to the service user in terms of time and distance.
*Availability* ^*1*^	Supply and demand	Resources available to meet the volume and needs of service users.
*Acceptability* ^*1*^	Consumer perception	Responsiveness to the attitudes, social norms and cultural values of service users.
*Affordability* ^*1*^	Financial and incidental costs	Costs incurred by both service providers and users.
*Adequacy* ^*1*^	Organisation	Organisation of the service (e.g., opening hours, referral or appointment systems, facility structures).
*Awareness* ^*2*^	Communication and information	Communication and information strategies enlisted to contact service users, including considerations of context and health literacy.

^1^The five dimensions of access as described by Penchansky and Thomas [28]

^2^The sixth dimension identified by Saurman [29]

Note, Table adapted from Saurman [29]

## Results

### Selection of sources of evidence

**Fig. 2:**
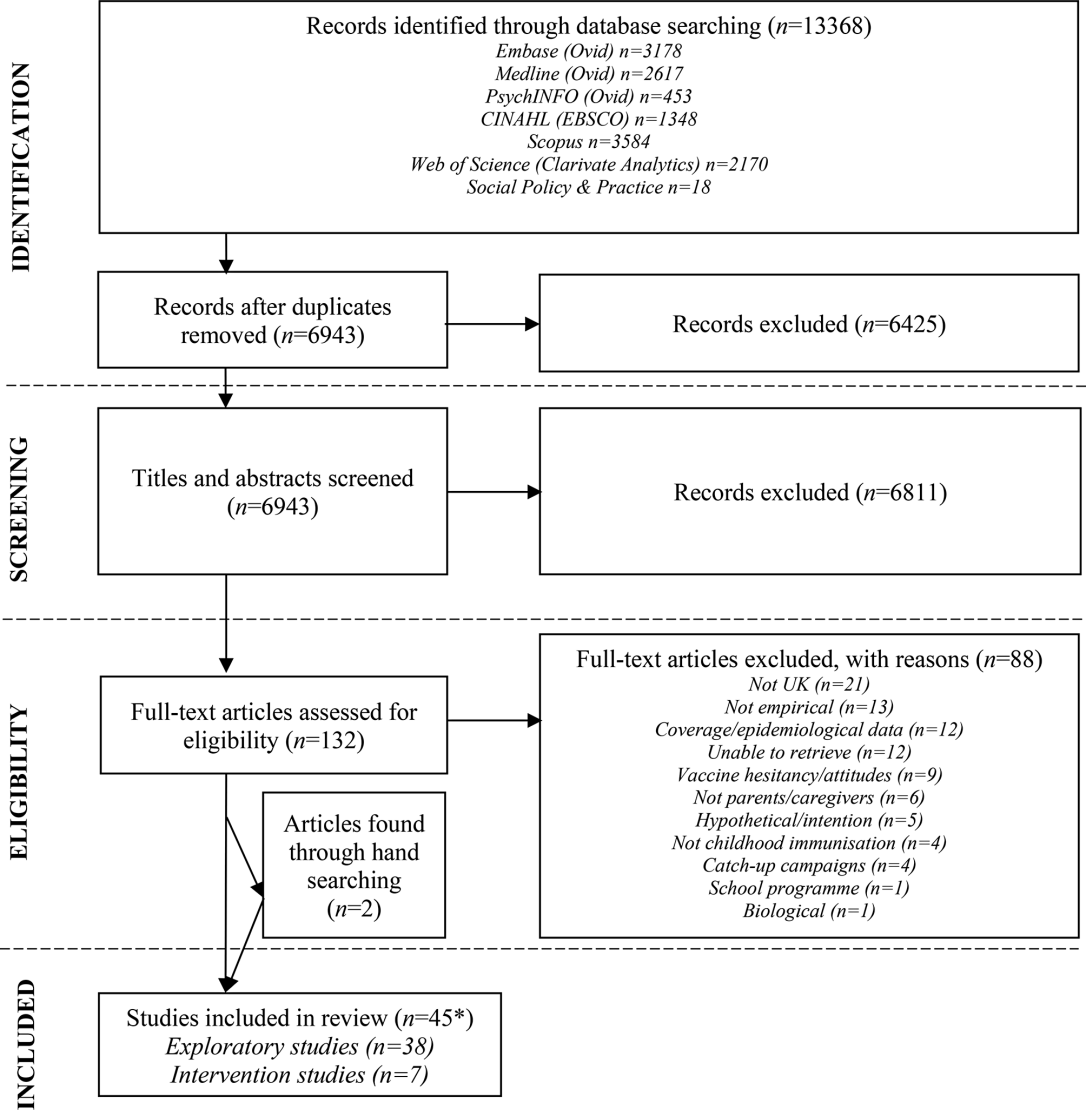
Flowchart of literature selection for the scoping review. *Note, the number of studies listed is 45, however two articles have been merged as they pertained to the same study [30, 31]

After the removal of duplicates, a total of 6943 articles were identified by database searching. Following the title and abstract screening of these articles, 132 articles remained. Following full-text screening, 43 studies were identified for inclusion. A further 2 were identified through hand searching of relevant reviews resulting in a total of 45. Most included studies were exploratory in nature (*n* = 38), with a minority being intervention related (*n* = 7). Figure 2 summarises the screening process, while the volume of results generated from the search strategies can be found within Additional File 1.

### Characteristics of included articles

The characteristics of the articles included in the review (on a per study basis) are summarised in a tabular format in Additional File 4.

### Conceptualising accessibility

A typology was created to represent the various ways accessibility was conceptualised within the studies included in the review. Five distinct types [1–5] were identified, the lower the number the more thorough the conceptualisation of access. The typology is presented in Table 5 alongside examples, study citations, and the prevalence of each type. Notably, the term ‘access’ or ‘accessibility’ was only used in 3/45 aim and objective statements.

**Table 5:** A typology of the conceptualisation of ‘accessibility’ within the literature

Type description	Example(s)	Exploratory studies	Intervention studies	Prevalence^1^
*1. Accessibility acknowledged within chosen theoretical framework*	• 5As Taxonomy for Determinants of Vaccine Uptake (Access, Affordability, Awareness, Acceptance and Activation) (Bell, 2019; Bell, 2020) • WHO Tailoring Immunisation Programmes (TIP) approach (Letley, 2018) • Social Ecological Model (SEM) (Jackson, 2016; 2017) • COM-B Model (Bell, 2021)	[30–35]	-	13% (*n* = 6/45)
*2. Distinguishes between accessibility/ practicality and attitudes /decision-making in-text*	• Johnson (2014, p873-874), ‘*However*,* this engagement was not described as ‘cognitive’ but rather as practical and contextual predicated by issues such as busyness*,* tiredness*,* ‘too much on their plate’. So it was the everyday*,* arguably mundane*,* processes and practices that the women in our focus group drew on to explain and describe their ‘choices’*’.	[36–45]	[46, 47)	27% (*n* = 12/45)
*3. Term ‘accessibility’ or ‘access’ used in passing or ambiguously with no clarification*	• Petts (2004, p11) writes that Asian mothers reported *‘low access’* to their General Practices but no further explanation or clarification was given.	[48–57]	[58]	24% (*n* = 11/45)
*4. No mention of ‘accessibility’ or ‘access’ despite findings*,* in part*,* including access related issues*	• Tomlinson (2013, p109) has no explicit focus on accessibility, as reflected in topic guide, only uses terms ‘*attitude’* and ‘*decision-making’* yet has important data on service responsiveness, and communication/information accessibility.	[59–69]	[70–73]	33% (*n* = 15/45)
*5. Claimed to consider only attitudinal constructs despite findings*,* in part*,* including access related issues*	• Gardner (2010, p220-221) distinguishes between ‘*motivational’* and ‘*organisational’* factors. They explicitly state that their paper assumes a ‘*motivational’* stance despite having data on communication/information accessibility.	[74, 75)	-	4% (*n* = 2/45)

1. Prevalence has been rounded to the nearest round number, resultantly the typologies do not sum to 100%

### Exploratory studies

#### Researching accessibility

**Fig. 3:**
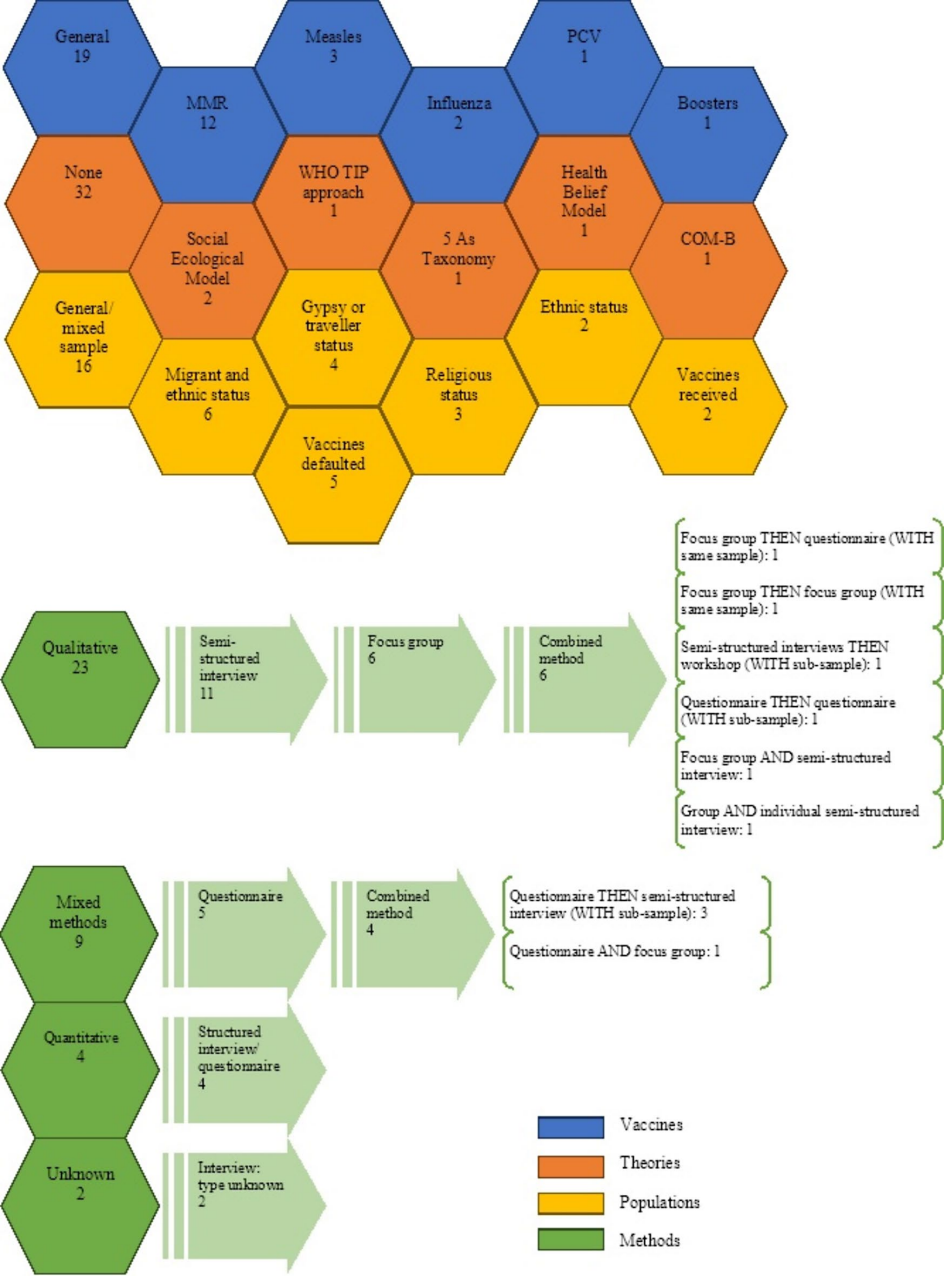
Visual representation of the vaccines, theories, populations, and methods of the studies included in the review

**Fig. 4:**
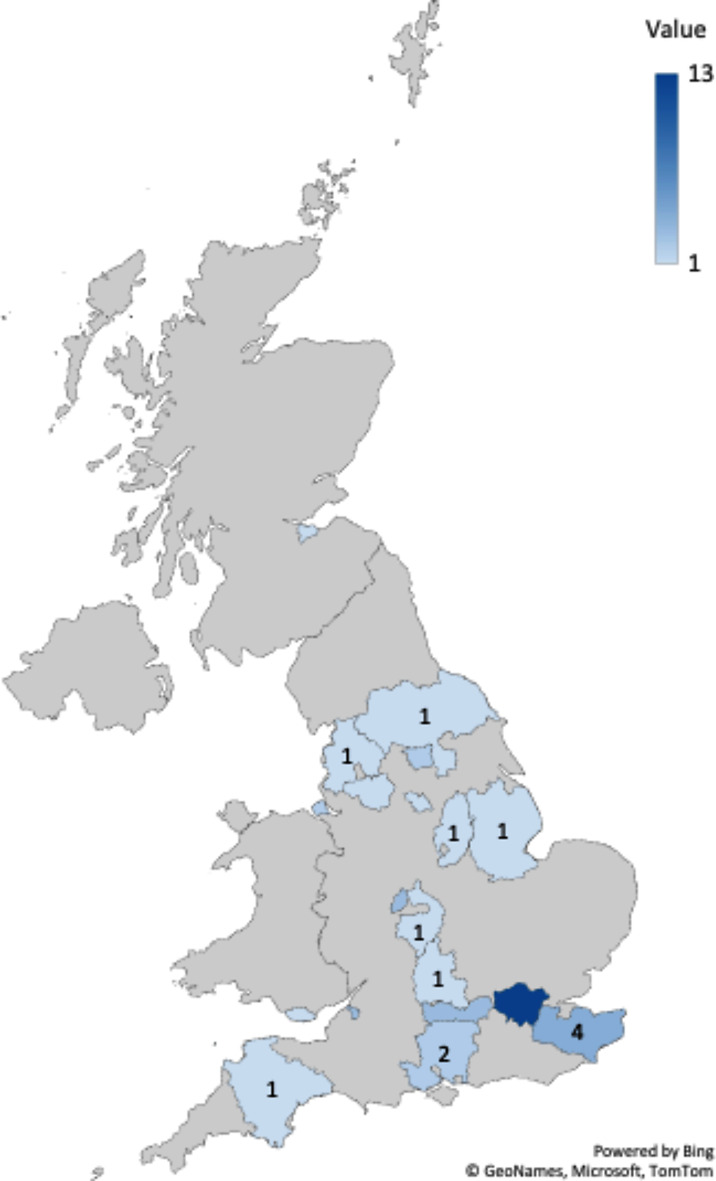
Visual representation of the geographical distribution of studies included in the review. Note, this is based on 32/38 studies due to lack of reporting (*n* = 5) and a nation-wide survey (*n* = 1). Some studies had multiple sites

An overview of the vaccines, theories, populations, and methods of the studies included in the review is presented in Fig. 3. From this, we can observe that exploring the childhood immunisation programme (*n* = 19) or MMR specifically (*n* = 12), without using a guiding theoretical framework (*n* = 32), within the general population (*n* = 16), using qualitative methodology (*n* = 23) were the most common approaches. Alternative approaches included focusing on groups identified as at risk of low uptake based upon migrant and ethnic status (*n* = 6); vaccine defaulter status (*n* = 5); Gypsy or traveller status (*n* = 4); religious status (*n* = 3); and ethnic status (*n* = 2). Only 2 studies looked at populations where vaccines had been received. Study coverage by geographical setting is represented in Fig. 4. The top research hubs were Greater London (*n* = 13), Kent (*n* = 4), Berkshire (*n* = 3), West Midlands (*n* = 3), and Bristol (*n* = 3).

### Accessibility findings

#### Parents’ experiences of accessing childhood vaccination services

Most of the studies did not exclusively focus on the ‘experience of accessing’ services and those which did (*n* = 17/38) often explored this dimension in passing. Positive experiences included healthcare workers (HCWs) who interacted and reassured parents or children, spent time discussing immunisation, and continuity of care [30, 31, 37, 60]. These experiences removed nerves during subsequent immunisation appointments, while negative experiences increased distrust and fear [34]. Some were happy to receive immunization reminders, while others interpreted this as pressure to comply [35, 40, 63, 64]. Similarly, some found immunisation emotionally difficult or anxiety inducing in terms of the child’s reaction, while others were able to turn it into an educational or learning experience [45, 62].

Negative experiences and dissatisfaction were cited with non-child-friendly facilities, unsympathetic treatment by clinic staff, and immunisation errors (e.g., repeat immunisation) [41, 45, 62]. Some studies reported that people felt that their concerns were a low priority or not taken seriously and in several cases this was cited as discrimination [30, 31, 34, 44, 61]. Having maternal or community knowledge dismissed meant some did not feel respected reducing satisfaction with the quality of care and HCW relations [30, 31, 35, 36]. Being rushed (i.e., time restrictions) also led to people feeling not listened to and left people with unaddressed needs, which in some cases led to tension and frustration [33, 55, 57]. Frustration was also reported with waiting times for appointments [30, 31]. Some felt practitioners were unwilling to engage in discussion of concerns, dismissive, condescending, or coercive [67]. Anticipation of experiencing conflict with health staff resulted in aversion to health service use [44].

### Childhood vaccination accessibility factors and recommendations

Using the six factors of accessibility developed by Saurman [25] as a guiding framework 18 concepts were identified. For example, within the accessibility (location) area of the framework two concepts were identified. Namely, the ‘locality of parents’ in terms of ease of getting to the clinic, and secondly the ‘locality of services’ in relation to the available choice of venues or outreach services. Three recommendations were identified, one of which was to protect funding for health visitors to enable outreach programmes to continue.

Of note, a further 8 concepts were identified which did not fit within the conceptual framework. These were parental factors which affected their ability to interact with immunisation services. This included housing status (nomadic, settled, moving house), competing interests (i.e., a ‘busy lifestyle’), prior experience, and English literacy. Recommendations were sparser for these concepts, but examples included improving temporary GP registration systems and ensuring new residence are up to date with their vaccinations. All concepts, sub-concepts and recommendations identified by the scoping review are presented in Table 6.

**Table 6:** Summary of accessibility concepts, sub-concepts, and recommendations for childhood immunisation services extracted from the studies included in the review

Concept	Sub-concepts	Recommendations
**Accessibility (location)**		
**Locality of parents**	(+/-) Ease of getting to the clinic. For example, isolated geographical locations, poorly served by public transport and no personal vehicle.	• On-site/local, immunisation outreach/drop-ins (in terms of information provision and vaccination). • Mult-agency forums (practitioners, parents, third parties) situated in nurseries could serve as community health information shops. • *Protect funding for health visitors.*
**Locality of services**	(+/-) Availability of immunisations services (choice of clinics and venues).	
	(+/-) Availability of outreach services.	
**Availability (supply and demand)**		
**Supply/demand of materials**	(-) Vaccines not offered, or only some available.	-
**Supply/demand of HCW time**	(-) Parents felt HCWs did not have time to relay immunisation information or discuss immunisation in detail.	-
	(+) Health visitors more accessible than GPs as information sources.	-
**Acceptability (consumer perception/responsiveness)**		
**Migration**	(-) Often migrant parents are not used to nurses issuing vaccination or being asked if the child is ‘well enough’ for vaccination, they expect this to be based upon their doctors’ assessment (also an issue of continuity of care). Vaccine programmes are also different, including choice of available formulations or brands (at a cost), and organisation of facilities (segregation of healthy and sick patients).	• Some migrants are unfamiliar with the UK health system, including nurses working in advanced roles in primary care. This needs to be addressed. • Continuity of care.
**Religion**	(-) Religious groups may experience anxiety that one of the MMR vaccines contains pig gelatine which is forbidden in Islam. (*Such groups should have access to Priorix instead of VaxPro*).	-
**Culture**	(-) Culturally unaware staff.	• HCWs to develop greater understanding of the communities they serve; cultural competence training for health professionals and frontline staff. • Named frontline person in GP practice to provide culturally respectful and supportive service. • *Protect funding for HCW training*
**Discrimination**	(-) Indirect and direct discrimination (denial of healthcare access).	• * Improve temporary GP registration systems.*
**Representation**	(+) Immunisers which match the population demographic.	• Employment of immunisation staff from communities as appropriate.
	-	• Parental meetings/interviews to improve vaccination process. • *More representation from community members in Clinical Commissioning Groups or local immunisation committee.*
**Affordability (financial and incidental costs)**		
**Actual cost**	(+) Vaccines are provided for free on the NHS.	-
	(-) Cost of travel to clinic.	-
The cost of *misinformation*	(-) If people are *unaware* that they are entitled to translators through the NHS they may seek their own interpreters who may be exploitative.	-
	(-) For migrants, *uncertainty* around access to free NHS services may reduce accessibility of the service.	-
**Adequacy (Organisation)**		
**External**	(+) Help from non-NHS-organisations (e.g., local councils).	-
**Process**	(-) Difficulties registering with GP (particularly for those with no proof of address, or birth certificate).	-
	(+/-) Usability and flexibility of booking systems, and availability of appointments (e.g., needing invitation letter to book).	• Have flexible and diverse systems for booking appointments.
	(-) Health professionals difficult to access due to formality and inaccessibility of the system (busy clinics or having to make an appointment).	-
	(-) If missed 6–8 week check difficult to get immunisation.	-
	-	• Identification of individual factors in health records (e.g., gypsy or traveller status) to enable tailored support and monitoring.
**Facility/provision**	(-) Facilities not child friendly (e.g., play areas, buggy storage).	-
	(+/-) Dissatisfactory appointment times; availability of extended opening hours or out of hours clinics (e.g., Sunday clinics).	-
	(+) Drop-in sessions and walk-in clinics.	• Drop-in immunisation.
	(+) Opportunistic vaccination.	• Opportunistic vaccination.
	(-) Long waiting times and facility overcrowding.	-
**Awareness (communication and information)**		
**Trustworthiness/ reliability**	(-) Lack of trustworthy information (including information on risks of vaccination) - perceived mainly due to financial interests of researchers, government and HCWs. However, conflicting advice was also cited. Mixed views on trustworthiness of HCWs/NHS.	• Realistic appraisal of risk (i.e., open and honest communication, safety data). • *Removal of target payments for vaccination to restore trust in information provision/communication.*
	(-) Overwhelming volume of information making it difficult to isolate and assess individual pieces of information.	-
	(+/-) Other parents and/or community members seen as trustworthy.	• Information campaigns to come from trusted sources which are separate to the UK Government (e.g., fellow parents, religious advisors).
	(-) HCWs could not answer questions or inaccurate information from HCWs (e.g., incorrect contraindication to immunisation).	• Ensure information provided by healthcare workers is accurate/consistent through *training* and regular updated for all healthcare professionals (e.g., valid contradictions). • *Protect funding for HCW training*
	(-) Miscommunication between health providers (i.e., local hospital and GP).	-
	-	• Dissemination of accurate information (e.g., how immunisation works, why it is important and valid contradictions to immunisation).
**Absence**	(-) Unaware of immunisation schedule or available solutions (e.g., walk-in clinics).	• Ensure mothers aware of service times and venues.
	(-) Information provided insufficient to address information needs (including no information provision at all, lack of what to expect post vaccination).	• Send leaflets with appointment cards. • Parental advice on how to deal with short term side-effects. • Parental advice on how to explain immunisation to their children. • More detailed factsheets available for parents desiring more information.
	(-) Lack of contact/verbal information with HCWs (HCWs perceived as too busy to ask questions).	• More timely/engaging information transfer – transfer in terms of seeing parents as more than passive recipients of information and enabling collaborative information exchange. • *Longer appointment slots.*
	(-) Lack of interpreters.	• *Increased access to interpreters or bilingual healthcare workers.* • Vaccination and broader health literature made available in translated forms.
	(-) Lack of tailored information provision (e.g., translated information, rashes on black skin), or lack of signposting to such information.	• Tailored communication. • Identification of individual factors in health records (e.g., gypsy or traveller status) to enable tailored support and monitoring.
**Methods**	(+) Community based channels of communication.	• Use local/community communication channels, including social media and magazines.
	(+) Vaccine reminders in the form of recall letters, reminder texts and telephone calls.	• Send leaflets with appointment cards.
	(+) Advertising and publicity efforts (e.g., TV, press, leaflets).	-
	(+) Verbal reminders give opportunity for discussion.	• More timely/engaging information transfer – transfer in terms of seeing parents as more than passive recipients of information and enabling collaborative information exchange. • Vaccination reminders given during health visitor appointments and general practice visits.
	(-) Inconsistency between and within practices in terms of vaccine reminders. Onus primarily on parents to book and remember appointments.	• A complete/prompt patient invite-reminder system that calls/recalls the child several times until the child receives immunisation which is flexible and diverse. • Provision of simple reminder aids (e.g., wall calendars, fridge magnets).
	(-) *Lower SES* groups found prevalence data difficult to understand and would find percentages more meaningful.	-
	(+/-) Simple immunisation information with pictures and clear explanations (particularly for *poor literacy*). NHS leaflets seen to be ‘dull’ and ‘uninformative’.	• Information provided using pictograms or pictures to help overcome literacy barriers.
**Experience of communication**	(-) Negative experiences with staff (e.g., unsympathetic, dismissive, coercive, lack of understanding or reassurance).	• HCWs should also focus on pastoral communication (i.e., listening, acknowledging concerns) in addition to providing ‘expert advice’.
**Other: does not fit in model**		
**-**	-	• Multisectoral working on cultural issues led by health professionals.
**Housing**	(-) A nomadic/travel-based lifestyle can result in reduced knowledge of local clinics, immunisation procedures, and missed appointments.	• HCWs to ask new residents about their vaccine history and record it and offer vaccinations to people unable to provide evidence of vaccination. • Discuss future travel/use of dual healthcare systems across countries to avoid missing or delaying vaccines. • * Improve temporary GP registration systems.*
	(+) Settled housing.	-
	(-) Moving house.	• HCWs to ask new residents about their vaccine history and record it and offer vaccinations to people unable to provide evidence of vaccination.
**Competing interests**	(-) Lack of time/energy (i.e., ‘busy lifestyle’) to find immunisation information alongside other immediate competing interests/priorities (e.g., work, long hours in low-paid jobs, other children). This includes considering immunisation and attending clinic appointments.	-
	(-) Forgetting.	• Provision of simple reminder aids (e.g., wall calendars, fridge magnets).
**Financial/ material status**	(-) Poverty and material needs (e.g., no access to car even if household has one, no money for taxi, lone mum/pregnant/other children, bus journeys not acceptable due to unreliable/infrequent/expensive/impractical).	• Reduce poverty.
**Illness**	(-) Some children are more likely to get ill due to living conditions or social environment which is a contradiction to immunisation (e.g., gypsy or traveller children).	-
	(-) Unable to attend practice due to illness.	-
**Information sources outside of health system provision**	(-) Use of media as source of information.	-
	(/) Consulting with social network for immunisation information (e.g., elders).	-
**Literacy/ education**	(-) Poor literacy (including not speaking English) or former education (e.g., understanding of immunisation and statistics). For example, not being able to read appointment letters so aware of next immunisation date. This also seems to affect the quality of the relationship parents report with HCWs.	• Raise educational outcomes. • *Recognise good practice with non-English speaking parents has resource implications.*
**Prior experience**	(-/+) Past experience(s), including those within the social group (e.g., long waiting times).	-
**Social support**	(-/+) Social isolation or social support (e.g., friends/family helping to register with GP, attend appointments).	• Increase social integration.
	(-) Appointment keeping not being part of cultural norm for some communities (e.g., G, R&T parents).	-
	(/) Increasing role of husbands/partners in immunisation.	-

Note, sub-concepts which improve, detract, or are neutral for accessibility are marked with ‘+’, ‘-‘, or ‘/’ respectively. Concepts which pertain to a specific demographic group are marked between two ‘*’ and in bold. Some concepts share similarities between different areas of the utilised accessibility framework; for example, lack of healthcare worker time is acknowledged as both a ‘supply and demand’ and ‘communication and information’ accessibility issue

**Fig. 5:**
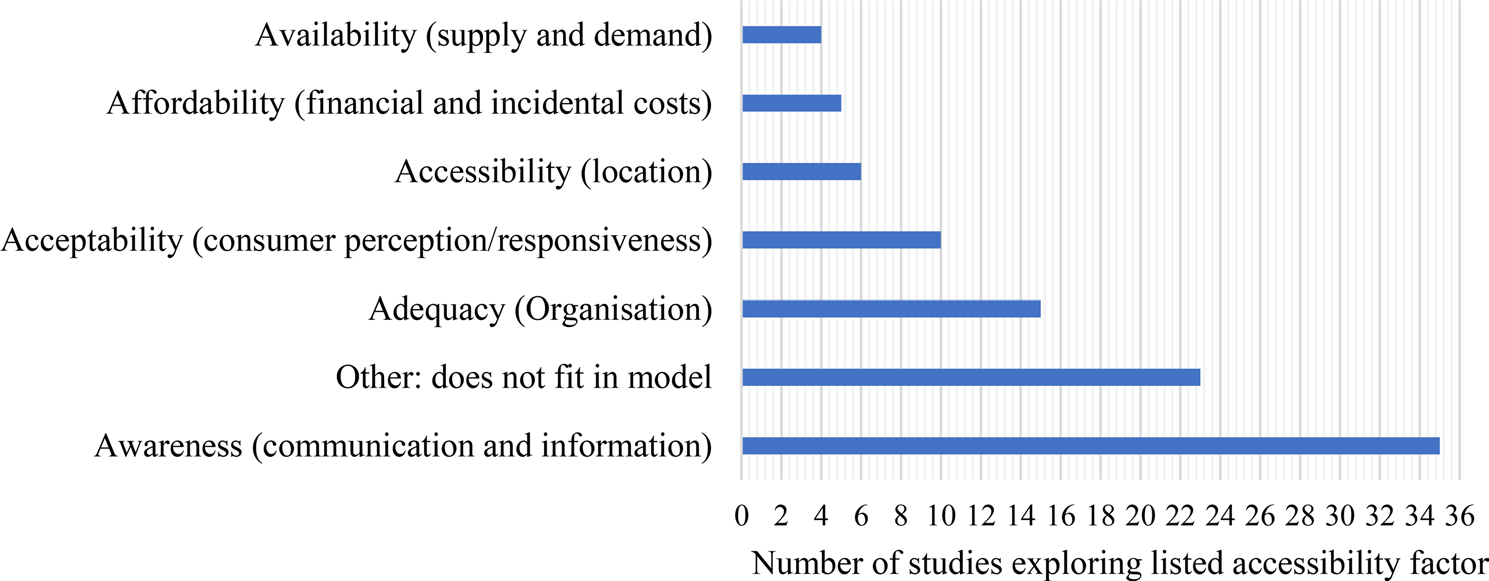
Exploration of accessibility factors based on prevalence across studies. Note, having a section indicated does not mean this factor was explored in-depth by the study (e.g., within the topic guide). For some papers, this may be only a sentence on the factor in question

The frequency with which studies explored various areas of the chosen accessibility framework is presented in Fig. 5. From this we can observe that the focus on access to information (92% of studies) was disproportionately large compared to other domains of accessibility such as availability (11%), affordability (13%), and proximity (i.e., location) (16%).

#### Intervention studies

Seven intervention studies met the inclusion criteria for this review, the most recent of which was published in 2016 [46, 47, 58, 70–73]. Of note, most studies (*n* = 5) sought to improve communication and information provision through the implementation of: a home record keeping tool; a call-recall system; a health education programme; a celebration card scheme; and a decision-aid intervention. The remaining studies targeted numerous components of the accessibility framework (*n* = 2) using: opportunistic inpatient vaccination; and a holistic health improvement initiative. Of the studies reporting intervention development (*n* = 5), methods included multidisciplinary group meetings, evidence reviews, identifying local best practice, and stakeholder workshops.

Study methods included randomised control trials (*n* = 2); pre-and post-intervention comparisons of uptake rates (*n* = 2); post-intervention uptake data (*n* = 1); quasi-experimental design (*n* = 1); and qualitative evaluation (*n* = 1). The reported results were: no impact (*n* = 3); increased vaccine uptake (*n* = 2); and unknown (*n* = 2). The interventions which reported increased uptake were the health education programme, and the informed-decision-making aid. Of note, studies reporting increased uptake were either looking at generalised uptake data unconnected to the study in the general population or applied an underpowered sub-analysis. The ‘unknown’ studies refer to a qualitative study (that explored suitability, feasibility and acceptability as opposed to effectiveness) and a study that looked at uptake with no control group.

#### Calls to research

Several recommendations for future research were identified, those which were highly specific to certain groups (e.g., Jewish and traveller groups) were omitted. Several recommendations were to do with ‘awareness’ and ‘communication of information’. This included how to best communicate potential vaccination risks so that vaccine information is deemed ‘trustworthy’ without overstating the risks or causing undue concern [55, 74]. Another suggested exploring how different socially situated groups receive and process health information [37]. Some stated a need to follow-up on the reported support from healthcare professionals when deviating from the vaccination programme and the propagation of misinformation [43, 56].

Beyond information provision, Carter and Jones [71] commented on the lack of active recall systems, with a significant minority of General Practices not actively encouraging immunisation, and that most parents had to take their own initiative for children to be vaccinated. Similarly, Conway [46] recommended that the importance of immunisation be emphasised at medical school and subsequent training. One study [47] recommended further exploration into the barriers to vaccine uptake such as being a lone-parent, or not being registered with a GP.

Two of the identified studies recommended further exploration of the dichotomy between culture or ethnicity and socioeconomic status, and that more research was needed to understand the importance of socioeconomic status independent of ethnicity [52, 55]. Another wanted to trace the paths and social processes surrounding vaccination [38]. It was put forward that views and practices needed to be monitored over time, that this was not a static phenomenon, and greater understanding of how and why vaccine uptake changes is central to informing future intervention [37].

## Discussion

Accessibility is poorly conceptualised within most of the research conducted on childhood immunisation uptake within the UK. This is evidenced by 61% of the articles included in this review using the term ‘access’ ambiguously with no further discussion (24%), not mentioning ‘access’ at all (33%), or claiming to take an attitudinal stance (4%), despite all discussing accessibility issues as according to the dimensions of access developed by Penchansky and Thomas [28] and Saurman [29]. While 27% distinguish between accessibility and attitudes in-text, this does not present a sufficient conceptual framework with which to guide future research.

We acknowledge that this, in part, is because exploring accessibility was not an explicitly objective of many of these studies. This is implied by the term ‘access’ or ‘accessibility’ only being used in 3/45 aim and objective statements. However, these were the only studies idented as part of a systematic scoping review of research with accessibility related data. This in turn demonstrates the need for both better conceptualisation and research priority on the accessibility of childhood vaccination services within the UK. Given the significant role accessibility is likely to play in vaccine uptake, unless focusing on a sample where vaccines have been delayed (by parental choice) or refused, then accessibility should be considered in tandem with vaccine hesitancy. Otherwise, researchers run the risk of limiting the scope of their findings based on their own conceptual ideas regarding the drivers of poor uptake rather than the lived reality of parents.

Smith and Newton [44] outline how *material* and *social* resources are core components of decision-making in relation immunisation. In other words, beyond believes regarding the vaccine or disease severity, the ramifications of ‘*day-to-day socio-material circumstances*’ shape decisions and experiences of vaccination services. Smith and Newton [44] note that the current emphasis on attitudes as opposed to accessibility is significantly flawed and that the difference between populations with high or low vaccine uptake is not willingness, but the drastically different context in which decisions are made. Placing causal power away from attitudes and towards context has dramatic consequences for research on vaccine uptake and the interventions which are predicated on them [44]. Notably, where vaccine hesitancy is the root cause, the main onus is on the decision-making of the parent. Interventions typically take the form of information provision. Contrastingly, where accessibility is the root cause, the main onus is on the service providers and interventions would have to address structural (rather than ideological) barriers to vaccine uptake.

This echoes the call to action made by New in 1991, in which the author criticised the Health Belief Model for turning a blind eye to the impact of social constraints and instead focusing on the belief structures of the individual. New and Senior [41] called for a more accurate model which moved away from the notion of *choice*, that was termed a *constraints-orientated model*. Many accessibility factors were identified as described across all six of the Dimensions of Access. As consistent with the over emphasis on cognition and decision making, information provision and communication dominated the research field being covered within 92% of exploratory studies, compared to 11–61% for all other factors. This is corroborated by the developers of the 5 A’s Taxonomy, which was based on a narrative review, which also cites that ‘acceptance’ was the most commonly studied aspect [76].

This should not be assumed as the irrelevance of these factors, as outlined previously many of these studies had no direct intention to explore access related issues and hence it can be inferred that for many studies these factors spontaneously emerged from participants despite topic guides having other agendas. Furthermore, there was no minimum threshold for ‘coverage of an issue’ so many of these studies may have only mentioned a factor in passing. None of the studies explicitly differentiated between factors of importance and the experience of service use. Despite this, a handful of studies did comment on this dimension in passing. Positive and negative experiences were very much to do with the accessibility of the services, particularly in relation to the accessibility of healthcare workers’ time to discuss immunisation due to both appointment length and willingness on the part of the practitioner.

Promisingly, 13% (*n* = 5) of studies used a guiding theoretical framework that acknowledged access constraints: the Social Ecological Model (SEM) [77]; the COM-B model [78] as adapted to vaccination [79], which also underpins the WHO Tailoring Immunisation Programmes (TIP) approach; and the 5As Taxonomy for Determinants of Vaccine Uptake [76]. While these frameworks include components which acknowledge access (e.g., ‘social’ or ‘physical’ opportunity within COM-B), it is unsurprising that alternative approaches which are built to explore ‘accessibility’ are more nuanced in their ability to define (and thus explore) the phenomenon. While accessibility models are not inherently better than those that take a more holistic approach, given the prevalence of barriers to vaccination there is scope for research which interrogates accessibility with greater conceptual depth. It is important to have a sufficient conceptual frame which considers all potential barriers, or this leaves ‘blind spots’ within the data collection and analysis [79].

The 5As Taxonomy, for instance, explicitly states how capturing such constraints is beyond its scope. The authors sought to create a taxonomy that could define all of the *non-*socio-demographic determinants of vaccine uptake. The reason cited for the exclusion of socio-demographic determinants was because ‘*these factors*,* while important*,* cannot be influenced by interventions*’ [76]. While the authors discuss using further research to weight the categories based on socio-cultural contexts, the omission of socio-demographic factors from the taxonomy leaves out a crucial piece of the puzzle as stated in numerous articles [2, 6, 7, 9, 10, 41, 44]. Notably, despite utilising an accessibility framework, all the factors identified by the review could not be placed within the selected framework. This was typically due to an over-representation of service-level factors, compared to parent-level factors which affect accessibility.

The dichotomy (and interface) between parental and service related factors is more extensively articulated in the accessibility framework devised by Levesque, Harris [80]. Access is seen as the result of the interface between the two. For instance, it is not only the cost of a service but also an individual’s ability to pay which determines access. Access is conceptualised along a patients journey from identifying health care needs through to health care utilisation and consequences. It comprises of five service-related factors and five corresponding abilities of people to interact with the service dimensions and generate access. This framework is credited as being one of the most comprehensive conceptualisations of healthcare access and is gaining acceptance among experts [81]. This may be of value when investiagting the accessibility of childhood vaccination services within the UK.

The recommendations provided within the literature are typically natural and practical in response to the issues listed. For example, for unsuitable opening hours, change the opening hours. Interestingly, a handful of articles go beyond the service (e.g., service provision, staff training, service funding) and discuss the role of the communities themselves. For example, studies that cited community-based information provision. One paper suggested using multi-agency forums situated in nurseries to act as immunisation information shops, another took this a step further and cited the need to reduce poverty, raise educational outcomes, and increase social integration. Beyond recommendations, only seven intervention studies were identified by the review. Furthermore, the vast majority of these (*n* = 5) were centred on communication and information. Moreover, the success recorded was limited or based upon limited evidence. More work is needed to devise and test feasible, appropriate, and effective interventions to increase the accessibility of childhood immunisation services within the UK.

### Limitations

This review sought to map the existing literature rather than evaluate the existing evidence as would have been the objective of a systematic review. Hence, as it is not possible to speak to the reliability or the quality of the factors extracted from the results sections of the included studies, it is not appropriate for these findings to inform intervention design without subjecting them to further empirical investigation. While there was a strong rationale for not including grey literature, it is important to acknowledge that work or learning taking place internally within the NHS may not be captured as a result. That said, even were the review to have included grey literature that was unlikely to report the methodological data needed, this would not have guaranteed capturing the tacit knowledge within the NHS as this is often embedded within organisational systems [82].

## Conclusions

Despite there being a call to action for greater focus on structural barriers to childhood immunisation within the UK, this issue remains lacking in conceptualisation and exploration. This, in part, is because exploring accessibility was not an explicit objective of many of the studies included in the review. While attitudes are undoubtedly important, parental context warrants greater attention and is often considered a peripheral factor rather than a central causal construct. Research informed by theoretical frames that clearly differentiate between parental attitude and accessibility is needed, including the dichotomy between accessibility based upon service provision (e.g., opening hours) and parental profile (e.g., journey to the clinic).

Recommended next steps within the literature included developing the research base on communication and information provision. Although important, they continue to emphasise an attitudinal perspective where, with more information, parents will choose to vaccinate rather than recognising that it may not be parental choice but parental opportunity that is key. Hence, greater gaps exist in relation to other calls to action, such as the reduced ability of some parents to prioritise vaccination, socioeconomic impacts, and the views and experiences of people through time. While service accessibility is part of the NHS vaccination strategy, research with greater focus and clarity on accessibility would shed light on how best to approach this target. 

## Electronic supplementary material

Below is the link to the electronic supplementary material.

Supplementary Material 1

Supplementary Material 2

Supplementary Material 3

Supplementary Material 4

## Data Availability

Data is provided within the manuscript or supplementary information files.
