# Supplementary material for: Parents’ and informal caregivers’ experiences of accessing childhood vaccination services within the United Kingdom: a systematic scoping review of empirical evidence
Source: BMC Public Health. 2024 Dec 18;24:3434. doi: 10.1186/s12889-024-20981-0 (PMC11653997; doi:10.1186/s12889-024-20981-0)
Supplement: Supplementary file 1 — Supplementary Material 1 [file 12889_2024_20981_MOESM1_ESM.docx]

**Additional File 1: Search Strategy**

**Embase Classic+Embase <1947 to 2022 January 26>**

| **#** | **Query** | **Results from 27 Jan 2022** |
| --- | --- | --- |
| 1 | (parent* or mother* or father* or caregiver* or "care giver*" or stepfather* or stepmother*).mp. | 1,108,574 |
| 2 | exp parent/ | 301,768 |
| 3 | single parent child/ | 18 |
| 4 | single-parent family/ | 311 |
| 5 | working mother/ | 691 |
| 6 | caregiver/ | 95,938 |
| 7 | parenthood/ | 4,442 |
| 8 | parental consent/ | 4,597 |
| 9 | parental behavior/ | 11,833 |
| 10 | or/1-9 | 1,139,369 |
| 11 | (experience* or access* or view* or belief* or opinion* or assessment* or attitude* or feeling* or idea* or impression* or judgement* or "point of view*" or perspective* or perception* or concern* or accept* or hesita* or refus* or abstain* or declin* or resit* or object* or deny* or denier* or decision*).mp. | 11,629,391 |
| 12 | personal experience/ | 55,007 |
| 13 | experience/ | 38,399 |
| 14 | health care access/ | 72,750 |
| 15 | or/11-14 | 11,629,391 |
| 16 | ((child* or infant* or newborn* or neonate* or postnatal or baby or babies or toddler*) and (immunis* or immuniz* or vaccin* or underimmunis* or underimmuniz* or undervaccinat* or DTaP or IPV or Hib or HepB or MenB or Rotavirus or PCV or MenC or MMR or LAIV or Engerix B or HBvaxPRO or Infanrix hexa or BCG)).mp. | 152,098 |
| 17 | child/ | 2,164,426 |
| 18 | childhood/ | 96,756 |
| 19 | infant/ | 755,459 |
| 20 | newborn/ | 653,933 |
| 21 | postnatal care/ | 8,243 |
| 22 | baby/ | 23,726 |
| 23 | toddler/ | 5,794 |
| 24 | immunization/ | 118,417 |
| 25 | active immunization/ | 9,090 |
| 26 | mass immunization/ | 4,687 |
| 27 | vaccination/ | 184,280 |
| 28 | vaccine/ | 78,355 |
| 29 | diphtheria pertussis tetanus vaccine/ | 9,084 |
| 30 | poliomyelitis vaccine/ | 11,097 |
| 31 | Haemophilus influenzae type b vaccine/ | 5,154 |
| 32 | hepatitis B vaccine/ | 20,441 |
| 33 | Meningococcus vaccine/ | 7,832 |
| 34 | Rotavirus vaccine/ | 6,106 |
| 35 | Pneumococcus vaccine/ | 21,513 |
| 36 | influenza vaccine/ | 42,559 |
| 37 | recombinant hepatitis B vaccine/ | 2,372 |
| 38 | diphtheria pertussis poliomyelitis tetanus Haemophilus influenzae type b hepatitis B vaccine/ | 592 |
| 39 | BCG vaccine/ | 46,344 |
| 40 | or/17-23 | 3,027,739 |
| 41 | or/24-39 | 418,483 |
| 42 | 40 and 41 | 72,213 |
| 43 | 16 or 42 | 152,098 |
| 44 | exp United Kingdom/ | 471,993 |
| 45 | (national health service* or nhs*).mp,in. | 396,598 |
| 46 | english.mp. | 153,578 |
| 47 | (gb or "g.b." or britain* or british* or uk or "u.k." or united kingdom* or england* or northern ireland* or northern irish* or scotland* or scottish* or wales or welsh*).mp,jw,in. | 4,099,169 |
| 48 | (bath or "bath's" or birmingham or "birmingham's" or bradford or "bradford's" or brighton or "brighton's" or bristol or "bristol's" or carlisle* or "carlisle's" or cambridge or "cambridge's" or canterbury or "canterbury's" or chelmsford or "chelmsford's" or chester or "chester's" or chichester or "chichester's" or coventry or "coventry's" or derby or "derby's" or durham or "durham's" or ely or "ely's" or exeter or "exeter's" or gloucester or "gloucester's" or hereford or "hereford's" or hull or "hull's" or lancaster or "lancaster's" or leeds* or leicester or "leicester's" or lincoln or "lincoln's" or liverpool or "liverpool's" or london or "london's" or manchester or "manchester's" or newcastle or "newcastle's" or norwich or "norwich's" or nottingham or "nottingham's" or oxford or "oxford's" or peterborough or "peterborough's" or plymouth or "plymouth's" or portsmouth or "portsmouth's" or preston or "preston's" or ripon or "ripon's" or salford or "salford's" or salisbury or "salisbury's" or sheffield or "sheffield's" or southampton or "southampton's" or st albans or stoke or "stoke's" or sunderland or "sunderland's" or truro or "truro's" or wakefield or "wakefield's" or wells or westminster or "westminster's" or winchester or "winchester's" or wolverhampton or "wolverhampton's" or worcester or "worcester's" or york or "york's").mp,in. | 4,266,322 |
| 49 | (bangor or "bangor's" or cardiff or "cardiff's" or newport or "newport's" or st asaph or "st asaph's" or st davids or swansea or "swansea's").mp,in. | 112,078 |
| 50 | (aberdeen or "aberdeen's" or dundee or "dundee's" or edinburgh or "edinburgh's" or glasgow or "glasgow's" or inverness or perth or "perth's" or stirling or "stirling's").mp,in. | 463,468 |
| 51 | (armagh or "armagh's" or belfast or "belfast's" or lisburn or "lisburn's" or londonderry or "londonderry's" or derry or "derry's" or newry or "newry's").mp,in. | 52,646 |
| 52 | or/44-51 | 6,359,367 |
| 53 | 10 and 15 and 43 and 52 | 3,178 |

**Ovid MEDLINE(R) ALL <1946 to January 26, 2022>**

| **#** | **Query** | **Results from 27 Jan 2022** |
| --- | --- | --- |
| 1 | (parent* or mother* or father* or caregiver* or "care giver*" or stepfather* or stepmother*).mp. | 822,279 |
| 2 | exp Parents/ | 128,476 |
| 3 | Single Parent/ | 1,229 |
| 4 | Single-Parent Family/ | 565 |
| 5 | Caregivers/ | 44,032 |
| 6 | Parenting/ | 19,224 |
| 7 | Parental Consent/ | 3,321 |
| 8 | Maternal Behavior/ | 11,959 |
| 9 | Paternal Behavior/ | 1,892 |
| 10 | or/1-9 | 826,088 |
| 11 | (experience* or access* or view* or belief* or opinion* or assessment* or attitude* or feeling* or idea* or impression* or judgement* or "point of view*" or perspective* or perception* or concern* or accept* or hesita* or refus* or abstain* or declin* or resit* or object* or deny* or denier* or decision*).mp. | 8,018,270 |
| 12 | exp Attitude/ | 616,775 |
| 13 | exp Emotions/ | 277,913 |
| 14 | Judgment/ | 20,327 |
| 15 | Perception/ | 40,622 |
| 16 | Decision Making/ | 101,419 |
| 17 | Health Services Accessibility/ | 82,275 |
| 18 | or/11-17 | 8,216,247 |
| 19 | ((child* or infant* or newborn* or neonate* or postnatal or baby or babies or toddler*) and (immunis* or immuniz* or vaccin* or underimmunis* or underimmuniz* or undervaccinat* or DTaP or IPV or Hib or HepB or MenB or Rotavirus or PCV or MenC or MMR or LAIV or Engerix B or HBvaxPRO or Infanrix hexa or BCG)).mp. | 117,320 |
| 20 | exp Child/ | 2,044,738 |
| 21 | exp Infant/ | 1,204,931 |
| 22 | Postnatal Care/ | 6,184 |
| 23 | Immunization/ | 52,545 |
| 24 | Immunization Schedule/ | 11,286 |
| 25 | exp Immunization Programs/ | 15,282 |
| 26 | exp Vaccination/ | 96,212 |
| 27 | Vaccines/ | 24,389 |
| 28 | Diphtheria-Tetanus-acellular Pertussis Vaccines/ | 1,341 |
| 29 | Poliovirus Vaccine, Inactivated/ | 3,068 |
| 30 | Haemophilus Vaccines/ | 3,095 |
| 31 | Hepatitis B Vaccines/ | 9,724 |
| 32 | Meningococcal Vaccines/ | 3,721 |
| 33 | Rotavirus Vaccines/ | 2,673 |
| 34 | Heptavalent Pneumococcal Conjugate Vaccine/ | 1,245 |
| 35 | Influenza Vaccines/ | 25,034 |
| 36 | BCG Vaccine/ | 19,893 |
| 37 | or/20-22 | 2,645,472 |
| 38 | or/23-36 | 210,267 |
| 39 | 37 and 38 | 50,781 |
| 40 | 19 or 39 | 117,320 |
| 41 | exp United Kingdom/ | 382,346 |
| 42 | (national health service* or nhs*).mp,in. | 237,781 |
| 43 | english.mp. | 1,599,968 |
| 44 | (gb or "g.b." or britain* or british* or uk or "u.k." or united kingdom* or england* or northern ireland* or northern irish* or scotland* or scottish* or wales or welsh*).mp,jw,in. | 2,729,351 |
| 45 | (bath or "bath's" or birmingham or "birmingham's" or bradford or "bradford's" or brighton or "brighton's" or bristol or "bristol's" or carlisle* or "carlisle's" or cambridge or "cambridge's" or canterbury or "canterbury's" or chelmsford or "chelmsford's" or chester or "chester's" or chichester or "chichester's" or coventry or "coventry's" or derby or "derby's" or durham or "durham's" or ely or "ely's" or exeter or "exeter's" or gloucester or "gloucester's" or hereford or "hereford's" or hull or "hull's" or lancaster or "lancaster's" or leeds* or leicester or "leicester's" or lincoln or "lincoln's" or liverpool or "liverpool's" or london or "london's" or manchester or "manchester's" or newcastle or "newcastle's" or norwich or "norwich's" or nottingham or "nottingham's" or oxford or "oxford's" or peterborough or "peterborough's" or plymouth or "plymouth's" or portsmouth or "portsmouth's" or preston or "preston's" or ripon or "ripon's" or salford or "salford's" or salisbury or "salisbury's" or sheffield or "sheffield's" or southampton or "southampton's" or st albans or stoke or "stoke's" or sunderland or "sunderland's" or truro or "truro's" or wakefield or "wakefield's" or wells or westminster or "westminster's" or winchester or "winchester's" or wolverhampton or "wolverhampton's" or worcester or "worcester's" or york or "york's").mp,in. | 2,554,247 |
| 46 | (bangor or "bangor's" or cardiff or "cardiff's" or newport or "newport's" or st asaph or "st asaph's" or st davids or swansea or "swansea's").mp,in. | 63,244 |
| 47 | (aberdeen or "aberdeen's" or dundee or "dundee's" or edinburgh or "edinburgh's" or glasgow or "glasgow's" or inverness or perth or "perth's" or stirling or "stirling's").mp,in. | 277,398 |
| 48 | (armagh or "armagh's" or belfast or "belfast's" or lisburn or "lisburn's" or londonderry or "londonderry's" or derry or "derry's" or newry or "newry's").mp,in. | 30,173 |
| 49 | or/41-48 | 5,617,257 |
| 50 | 10 and 18 and 40 and 49 | 2,617 |

**APA PsycInfo <1806 to January Week 4 2022>**

| **#** | **Query** | **Results from 28 Jan 2022** |
| --- | --- | --- |
| 1 | (parent* or mother* or father* or caregiver* or "care giver*" or stepfather* or stepmother*).mp. | 461,340 |
| 2 | exp parents/ | 126,406 |
| 3 | caregivers/ | 32,410 |
| 4 | parenting/ | 12,824 |
| 5 | or/1-4 | 461,493 |
| 6 | (experience* or access* or view* or belief* or opinion* or assessment* or attitude* or feeling* or idea* or impression* or judgement* or "point of view*" or perspective* or perception* or concern* or accept* or hesita* or refus* or abstain* or declin* or resit* or object* or deny* or denier* or decision*).mp. | 3,019,123 |
| 7 | health care access/ | 1,685 |
| 8 | attitudes/ | 29,161 |
| 9 | emotions/ | 52,094 |
| 10 | judgment/ | 25,370 |
| 11 | decision making/ | 80,566 |
| 12 | or/6-11 | 3,036,291 |
| 13 | ((child* or infant* or newborn* or neonate* or postnatal or baby or babies or toddler*) and (immunis* or immuniz* or vaccin* or underimmunis* or underimmuniz* or undervaccinat* or DTaP or IPV or Hib or HepB or MenB or Rotavirus or PCV or MenC or MMR or LAIV or Engerix B or HBvaxPRO or Infanrix hexa or BCG)).mp. | 5,624 |
| 14 | neonatal period/ | 1,850 |
| 15 | postnatal period/ | 5,583 |
| 16 | child health/ | 455 |
| 17 | immunization/ | 5,294 |
| 18 | or/14-16 | 7,855 |
| 19 | 17 and 18 | 18 |
| 20 | 13 or 19 | 5,624 |
| 21 | (national health service* or nhs*).mp,in. | 27,132 |
| 22 | english.mp. | 147,450 |
| 23 | (gb or "g.b." or britain* or british* or uk or "u.k." or united kingdom* or england* or northern ireland* or northern irish* or scotland* or scottish* or wales or welsh*).mp,jw,in. | 552,396 |
| 24 | (bath or "bath's" or birmingham or "birmingham's" or bradford or "bradford's" or brighton or "brighton's" or bristol or "bristol's" or carlisle* or "carlisle's" or cambridge or "cambridge's" or canterbury or "canterbury's" or chelmsford or "chelmsford's" or chester or "chester's" or chichester or "chichester's" or coventry or "coventry's" or derby or "derby's" or durham or "durham's" or ely or "ely's" or exeter or "exeter's" or gloucester or "gloucester's" or hereford or "hereford's" or hull or "hull's" or lancaster or "lancaster's" or leeds* or leicester or "leicester's" or lincoln or "lincoln's" or liverpool or "liverpool's" or london or "london's" or manchester or "manchester's" or newcastle or "newcastle's" or norwich or "norwich's" or nottingham or "nottingham's" or oxford or "oxford's" or peterborough or "peterborough's" or plymouth or "plymouth's" or portsmouth or "portsmouth's" or preston or "preston's" or ripon or "ripon's" or salford or "salford's" or salisbury or "salisbury's" or sheffield or "sheffield's" or southampton or "southampton's" or st albans or stoke or "stoke's" or sunderland or "sunderland's" or truro or "truro's" or wakefield or "wakefield's" or wells or westminster or "westminster's" or winchester or "winchester's" or wolverhampton or "wolverhampton's" or worcester or "worcester's" or york or "york's").mp,in. | 682,403 |
| 25 | (bangor or "bangor's" or cardiff or "cardiff's" or newport or "newport's" or st asaph or "st asaph's" or st davids or swansea or "swansea's").mp,in. | 20,219 |
| 26 | (aberdeen or "aberdeen's" or dundee or "dundee's" or edinburgh or "edinburgh's" or glasgow or "glasgow's" or inverness or perth or "perth's" or stirling or "stirling's").mp,in. | 76,608 |
| 27 | (armagh or "armagh's" or belfast or "belfast's" or lisburn or "lisburn's" or londonderry or "londonderry's" or derry or "derry's" or newry or "newry's").mp,in. | 6,393 |
| 28 | or/21-27 | 1,050,211 |
| 29 | 5 and 12 and 20 and 28 | 453 |

**CINAHL (EBSCO)**

| **#** | **Query** | **Limiters/Expanders** | **Last Run Via** | **Results from 28 Jan 2022** |
| --- | --- | --- | --- | --- |
| S50 | S6 AND S19 AND S40 AND S49 | Expanders - Apply equivalent subjects Search modes - Boolean/Phrase | Interface - EBSCOhost Research Databases Search Screen - Advanced Search Database - CINAHL Complete | 1,348 |
| S49 | S41 OR S42 OR S43 OR S44 OR S45 OR S46 OR S47 OR S48 | Expanders - Apply equivalent subjects Search modes - Boolean/Phrase | Interface - EBSCOhost Research Databases Search Screen - Advanced Search Database - CINAHL Complete | 1,163,872 |
| S48 | armagh or "armagh's" or belfast or "belfast's" or lisburn or "lisburn's" or londonderry or "londonderry's" or derry or "derry's" or newry or "newry's" | Expanders - Apply equivalent subjects Search modes - Boolean/Phrase | Interface - EBSCOhost Research Databases Search Screen - Advanced Search Database - CINAHL Complete | 1,134 |
| S47 | aberdeen or "aberdeen's" or dundee or "dundee's" or edinburgh or "edinburgh's" or glasgow or "glasgow's" or inverness or perth or "perth's" or stirling or "stirling's" | Expanders - Apply equivalent subjects Search modes - Boolean/Phrase | Interface - EBSCOhost Research Databases Search Screen - Advanced Search Database - CINAHL Complete | 22,821 |
| S46 | bangor or "bangor's" or cardiff or "cardiff's" or newport or "newport's" or “st asaph” or "st asaph's" or “st davids” or swansea or "swansea's" | Expanders - Apply equivalent subjects Search modes - Boolean/Phrase | Interface - EBSCOhost Research Databases Search Screen - Advanced Search Database - CINAHL Complete | 1,800 |
| S45 | bath or "bath's" or birmingham or "birmingham's" or bradford or "bradford's" or brighton or "brighton's" or bristol or "bristol's" or carlisle* or "carlisle's" or cambridge or "cambridge's" or canterbury or "canterbury's" or chelmsford or "chelmsford's" or chester or "chester's" or chichester or "chichester's" or coventry or "coventry's" or derby or "derby's" or durham or "durham's" or ely or "ely's" or exeter or "exeter's" or gloucester or "gloucester's" or hereford or "hereford's" or hull or "hull's" or lancaster or "lancaster's" or leeds* or leicester or "leicester's" or lincoln or "lincoln's" or liverpool or "liverpool's" or london or "london's" or manchester or "manchester's" or newcastle or "newcastle's" or norwich or "norwich's" or nottingham or "nottingham's" or oxford or "oxford's" or peterborough or "peterborough's" or plymouth or "plymouth's" or portsmouth or "portsmouth's" or preston or "preston's" or ripon or "ripon's" or salford or "salford's" or salisbury or "salisbury's" or sheffield or "sheffield's" or southampton or "southampton's" or “st albans” or stoke or "stoke's" or sunderland or "sunderland's" or truro or "truro's" or wakefield or "wakefield's" or wells or westminster or "westminster's" or winchester or "winchester's" or wolverhampton or "wolverhampton's" or worcester or "worcester's" or york or "york's" | Expanders - Apply equivalent subjects Search modes - Boolean/Phrase | Interface - EBSCOhost Research Databases Search Screen - Advanced Search Database - CINAHL Complete | 695,023 |
| S44 | gb or "g.b." or britain* or british* or uk or "u.k." or “united kingdom*” or england* or “northern ireland*” or “northern irish*” or scotland* or scottish* or wales or welsh* | Expanders - Apply equivalent subjects Search modes - Boolean/Phrase | Interface - EBSCOhost Research Databases Search Screen - Advanced Search Database - CINAHL Complete | 450,529 |
| S43 | english | Expanders - Apply equivalent subjects Search modes - Boolean/Phrase | Interface - EBSCOhost Research Databases Search Screen - Advanced Search Database - CINAHL Complete | 59,784 |
| S42 | “national health service*” or nhs* | Expanders - Apply equivalent subjects Search modes - Boolean/Phrase | Interface - EBSCOhost Research Databases Search Screen - Advanced Search Database - CINAHL Complete | 61,802 |
| S41 | (MH "United Kingdom+") | Expanders - Apply equivalent subjects Search modes - Boolean/Phrase | Interface - EBSCOhost Research Databases Search Screen - Advanced Search Database - CINAHL Complete | 333,170 |
| S40 | S20 OR S39 | Expanders - Apply equivalent subjects Search modes - Boolean/Phrase | Interface - EBSCOhost Research Databases Search Screen - Advanced Search Database - CINAHL Complete | 32,701 |
| S39 | S37 AND S38 | Expanders - Apply equivalent subjects Search modes - Boolean/Phrase | Interface - EBSCOhost Research Databases Search Screen - Advanced Search Database - CINAHL Complete | 16,699 |
| S38 | S23 OR S24 OR S25 OR S26 OR S27 OR S28 OR S29 OR S30 OR S31 OR S32 OR S33 OR S34 OR S35 OR S36 | Expanders - Apply equivalent subjects Search modes - Boolean/Phrase | Interface - EBSCOhost Research Databases Search Screen - Advanced Search Database - CINAHL Complete | 52,730 |
| S37 | S21 OR S22 | Expanders - Apply equivalent subjects Search modes - Boolean/Phrase | Interface - EBSCOhost Research Databases Search Screen - Advanced Search Database - CINAHL Complete | 730,351 |
| S36 | (MH "Measles-Mumps-Rubella Vaccine") | Expanders - Apply equivalent subjects Search modes - Boolean/Phrase | Interface - EBSCOhost Research Databases Search Screen - Advanced Search Database - CINAHL Complete | 1,974 |
| S35 | (MH "BCG Vaccine") | Expanders - Apply equivalent subjects Search modes - Boolean/Phrase | Interface - EBSCOhost Research Databases Search Screen - Advanced Search Database - CINAHL Complete | 1,692 |
| S34 | (MH "Influenza Vaccine") | Expanders - Apply equivalent subjects Search modes - Boolean/Phrase | Interface - EBSCOhost Research Databases Search Screen - Advanced Search Database - CINAHL Complete | 11,094 |
| S33 | (MH "Pneumococcal Vaccine") | Expanders - Apply equivalent subjects Search modes - Boolean/Phrase | Interface - EBSCOhost Research Databases Search Screen - Advanced Search Database - CINAHL Complete | 3,185 |
| S32 | (MH "Rotavirus Vaccines") | Expanders - Apply equivalent subjects Search modes - Boolean/Phrase | Interface - EBSCOhost Research Databases Search Screen - Advanced Search Database - CINAHL Complete | 809 |
| S31 | (MH "Meningococcal Vaccines") | Expanders - Apply equivalent subjects Search modes - Boolean/Phrase | Interface - EBSCOhost Research Databases Search Screen - Advanced Search Database - CINAHL Complete | 747 |
| S30 | (MH "Hepatitis B Vaccines") | Expanders - Apply equivalent subjects Search modes - Boolean/Phrase | Interface - EBSCOhost Research Databases Search Screen - Advanced Search Database - CINAHL Complete | 2,452 |
| S29 | (MH "HIB Vaccine") | Expanders - Apply equivalent subjects Search modes - Boolean/Phrase | Interface - EBSCOhost Research Databases Search Screen - Advanced Search Database - CINAHL Complete | 719 |
| S28 | (MH "Poliovirus Vaccine, Inactivated") | Expanders - Apply equivalent subjects Search modes - Boolean/Phrase | Interface - EBSCOhost Research Databases Search Screen - Advanced Search Database - CINAHL Complete | 276 |
| S27 | (MH "Diphtheria-Tetanus-acellular Pertussis Vaccines") | Expanders - Apply equivalent subjects Search modes - Boolean/Phrase | Interface - EBSCOhost Research Databases Search Screen - Advanced Search Database - CINAHL Complete | 479 |
| S26 | (MH "Vaccines") | Expanders - Apply equivalent subjects Search modes - Boolean/Phrase | Interface - EBSCOhost Research Databases Search Screen - Advanced Search Database - CINAHL Complete | 9,377 |
| S25 | (MH "Immunization Programs") | Expanders - Apply equivalent subjects Search modes - Boolean/Phrase | Interface - EBSCOhost Research Databases Search Screen - Advanced Search Database - CINAHL Complete | 6,299 |
| S24 | (MH "Immunization Schedule") | Expanders - Apply equivalent subjects Search modes - Boolean/Phrase | Interface - EBSCOhost Research Databases Search Screen - Advanced Search Database - CINAHL Complete | 3,290 |
| S23 | (MH "Immunization") | Expanders - Apply equivalent subjects Search modes - Boolean/Phrase | Interface - EBSCOhost Research Databases Search Screen - Advanced Search Database - CINAHL Complete | 28,077 |
| S22 | (MH "Postnatal Period") | Expanders - Apply equivalent subjects Search modes - Boolean/Phrase | Interface - EBSCOhost Research Databases Search Screen - Advanced Search Database - CINAHL Complete | 10,811 |
| S21 | (MH "Child+") | Expanders - Apply equivalent subjects Search modes - Boolean/Phrase | Interface - EBSCOhost Research Databases Search Screen - Advanced Search Database - CINAHL Complete | 721,755 |
| S20 | (child* or infant* or newborn* or neonate* or postnatal or baby or babies or toddler*) and (immunis* or immuniz* or vaccin* or underimmunis* or underimmuniz* or undervaccinat* or DTaP or IPV or Hib or HepB or MenB or Rotavirus or PCV or MenC or MMR or LAIV or “Engerix B” or HBvaxPRO or “Infanrix hexa” or BCG) | Expanders - Apply equivalent subjects Search modes - Boolean/Phrase | Interface - EBSCOhost Research Databases Search Screen - Advanced Search Database - CINAHL Complete | 32,701 |
| S19 | S7 OR S8 OR S9 OR S10 OR S11 OR S12 OR S13 OR S14 OR S15 OR S16 OR S17 OR S18 | Expanders - Apply equivalent subjects Search modes - Boolean/Phrase | Interface - EBSCOhost Research Databases Search Screen - Advanced Search Database - CINAHL Complete | 2,798,172 |
| S18 | (MH "Health Services Accessibility+") | Expanders - Apply equivalent subjects Search modes - Boolean/Phrase | Interface - EBSCOhost Research Databases Search Screen - Advanced Search Database - CINAHL Complete | 97,231 |
| S17 | (MH "Decision Making+") | Expanders - Apply equivalent subjects Search modes - Boolean/Phrase | Interface - EBSCOhost Research Databases Search Screen - Advanced Search Database - CINAHL Complete | 139,239 |
| S16 | (MH "Judgment") | Expanders - Apply equivalent subjects Search modes - Boolean/Phrase | Interface - EBSCOhost Research Databases Search Screen - Advanced Search Database - CINAHL Complete | 6,456 |
| S15 | (MH "Emotions+") | Expanders - Apply equivalent subjects Search modes - Boolean/Phrase | Interface - EBSCOhost Research Databases Search Screen - Advanced Search Database - CINAHL Complete | 157,695 |
| S14 | (MH "Parental Attitudes+") | Expanders - Apply equivalent subjects Search modes - Boolean/Phrase | Interface - EBSCOhost Research Databases Search Screen - Advanced Search Database - CINAHL Complete | 23,026 |
| S13 | (MH "Attitude of Health Personnel") | Expanders - Apply equivalent subjects Search modes - Boolean/Phrase | Interface - EBSCOhost Research Databases Search Screen - Advanced Search Database - CINAHL Complete | 49,382 |
| S12 | (MH "Attitude to Vaccines") | Expanders - Apply equivalent subjects Search modes - Boolean/Phrase | Interface - EBSCOhost Research Databases Search Screen - Advanced Search Database - CINAHL Complete | 1,386 |
| S11 | (MH "Caregiver Attitudes") | Expanders - Apply equivalent subjects Search modes - Boolean/Phrase | Interface - EBSCOhost Research Databases Search Screen - Advanced Search Database - CINAHL Complete | 2,957 |
| S10 | (MH "Attitude to Health") | Expanders - Apply equivalent subjects Search modes - Boolean/Phrase | Interface - EBSCOhost Research Databases Search Screen - Advanced Search Database - CINAHL Complete | 47,457 |
| S9 | (MH "Attitude") | Expanders - Apply equivalent subjects Search modes - Boolean/Phrase | Interface - EBSCOhost Research Databases Search Screen - Advanced Search Database - CINAHL Complete | 17,719 |
| S8 | (MH "Health Services Accessibility") | Expanders - Apply equivalent subjects Search modes - Boolean/Phrase | Interface - EBSCOhost Research Databases Search Screen - Advanced Search Database - CINAHL Complete | 95,955 |
| S7 | experience* or access* or view* or belief* or opinion* or assessment* or attitude* or feeling* or idea* or impression* or judgement* or "point of view*" or perspective* or perception* or concern* or accept* or hesita* or refus* or abstain* or declin* or resit* or object* or deny* or denier* or decision* | Expanders - Apply equivalent subjects Search modes - Boolean/Phrase | Interface - EBSCOhost Research Databases Search Screen - Advanced Search Database - CINAHL Complete | 2,738,058 |
| S6 | S1 OR S2 OR S3 OR S4 OR S5 | Expanders - Apply equivalent subjects Search modes - Boolean/Phrase | Interface - EBSCOhost Research Databases Search Screen - Advanced Search Database - CINAHL Complete | 360,911 |
| S5 | (MH "Parental Behavior") | Expanders - Apply equivalent subjects Search modes - Boolean/Phrase | Interface - EBSCOhost Research Databases Search Screen - Advanced Search Database - CINAHL Complete | 2,322 |
| S4 | (MH "Parenting") | Expanders - Apply equivalent subjects Search modes - Boolean/Phrase | Interface - EBSCOhost Research Databases Search Screen - Advanced Search Database - CINAHL Complete | 20,032 |
| S3 | (MH "Caregivers") | Expanders - Apply equivalent subjects Search modes - Boolean/Phrase | Interface - EBSCOhost Research Databases Search Screen - Advanced Search Database - CINAHL Complete | 39,343 |
| S2 | (MH "Parents+") | Expanders - Apply equivalent subjects Search modes - Boolean/Phrase | Interface - EBSCOhost Research Databases Search Screen - Advanced Search Database - CINAHL Complete | 103,282 |
| S1 | parent* or mother* or father* or caregiver* or "care giver*" or stepfather* or stepmother* | Expanders - Apply equivalent subjects Search modes - Boolean/Phrase | Interface - EBSCOhost Research Databases Search Screen - Advanced Search Database - CINAHL Complete | 360,010 |

**Scopus**

| **#** | **Query** | **Results from 28 Jan 2022** |
| --- | --- | --- |
| 5 | #1 AND #2 AND #3 AND #4 | 3,584 |
| 4 | TITLE-ABS-KEY ( {national health service*} OR nhs* OR english OR gb OR {g.b.} OR britain* OR british* OR uk OR {u.k.} OR {united kingdom*} OR england* OR {northern ireland*} OR {northern irish*} OR scotland* OR scottish* OR wales OR welsh* OR bath OR {bath's} OR birmingham OR {birmingham's} OR bradford OR {bradford's} OR brighton OR {brighton's} OR bristol OR {bristol's} OR carlisle* OR {carlisle's} OR cambridge OR {cambridge's} OR canterbury OR {canterbury's} OR chelmsford OR {chelmsford's} OR chester OR {chester's} OR chichester OR {chichester's} OR coventry OR {coventry's} OR derby OR {derby's} OR durham OR {durham's} OR ely OR {ely's} OR exeter OR {exeter's} OR gloucester OR {gloucester's} OR hereford OR {hereford's} OR hull OR {hull's} OR lancaster OR {lancaster's} OR leeds* OR leicester OR {leicester's} OR lincoln OR {lincoln's} OR liverpool OR {liverpool's} OR london OR {london's} OR manchester OR {manchester's} OR newcastle OR {newcastle's} OR norwich OR {norwich's} OR nottingham OR {nottingham's} OR oxford OR {oxford's} OR peterborough OR {peterborough's} OR plymouth OR {plymouth's} OR portsmouth OR {portsmouth's} OR preston OR {preston's} OR ripon OR {ripon's} OR salford OR {salford's} OR salisbury OR {salisbury's} OR sheffield OR {sheffield's} OR southampton OR {southampton's} OR {st albans} OR stoke OR {stoke's} OR sunderland OR {sunderland's} OR truro OR {truro's} OR wakefield OR {wakefield's} OR wells OR westminster OR {westminster's} OR winchester OR {winchester's} OR wolverhampton OR {wolverhampton's} OR worcester OR {worcester's} OR york OR {york's} OR bangor OR {bangor's} OR cardiff OR {cardiff's} OR newport OR {newport's} OR {st asaph} OR {st asaph's} OR {st davids} OR swansea OR {swansea's} OR aberdeen OR {aberdeen's} OR dundee OR {dundee's} OR edinburgh OR {edinburgh's} OR glasgow OR {glasgow's} OR inverness OR perth OR {perth's} OR stirling OR {stirling's} OR armagh OR {armagh's} OR belfast OR {belfast's} OR lisburn OR {lisburn's} OR londonderry OR {londonderry's} OR derry OR {derry's} OR newry OR {newry's} ) | 12,682,868 |
| 3 | TITLE-ABS-KEY ( ( child* OR infant* OR newborn* OR neonate* OR postnatal OR baby OR babies OR toddler* ) AND ( immunis* OR immuniz* OR vaccin* OR underimmunis* OR underimmuniz* OR undervaccinat* OR dtap OR ipv OR hib OR hepb OR menb OR rotavirus OR pcv OR menc OR mmr OR laiv OR {engerix b} OR hbvaxpro OR {infanrix hexa} OR bcg ) ) | 148,745 |
| 2 | TITLE-ABS-KEY ( experience* OR access* OR view* OR belief* OR opinion* OR assessment* OR attitude* OR feeling* OR idea* OR impression* OR judgement* OR {point of view*} OR perspective* OR perception* OR concern* OR accept* OR hesita* OR refus* OR abstain* OR declin* OR resit* OR object* OR deny* OR denier* OR decision* ) | 20,507,669 |
| 1 | TITLE-ABS-KEY ( parent* OR mother* OR father* OR caregiver* OR {care giver*} OR stepfather* OR stepmother* ) | 1,375,657 |

**Web of Science**

| **#** | **Query** | **Results from 28 Jan 2022** |
| --- | --- | --- |
| 5 | **#4 AND #3 AND #2 AND #1** | 2,170 |
| 4 | TS=(“national health service*” or nhs* or English or gb or "g.b." or britain* or british* or uk or "u.k." or “united kingdom*” or england* or “northern ireland*” or “northern irish*” or scotland* or scottish* or wales or welsh* or bath or "bath's" or birmingham or "birmingham's" or bradford or "bradford's" or brighton or "brighton's" or bristol or "bristol's" or carlisle* or "carlisle's" or cambridge or "cambridge's" or canterbury or "canterbury's" or chelmsford or "chelmsford's" or chester or "chester's" or chichester or "chichester's" or coventry or "coventry's" or derby or "derby's" or durham or "durham's" or ely or "ely's" or exeter or "exeter's" or gloucester or "gloucester's" or hereford or "hereford's" or hull or "hull's" or lancaster or "lancaster's" or leeds* or leicester or "leicester's" or lincoln or "lincoln's" or liverpool or "liverpool's" or london or "london's" or manchester or "manchester's" or newcastle or "newcastle's" or norwich or "norwich's" or nottingham or "nottingham's" or oxford or "oxford's" or peterborough or "peterborough's" or plymouth or "plymouth's" or portsmouth or "portsmouth's" or preston or "preston's" or ripon or "ripon's" or salford or "salford's" or salisbury or "salisbury's" or sheffield or "sheffield's" or southampton or "southampton's" or “st albans” or stoke or "stoke's" or sunderland or "sunderland's" or truro or "truro's" or wakefield or "wakefield's" or wells or westminster or "westminster's" or winchester or "winchester's" or wolverhampton or "wolverhampton's" or worcester or "worcester's" or york or "york's" or bangor or "bangor's" or cardiff or "cardiff's" or newport or "newport's" or “st asaph” or "st asaph's" or “st davids” or swansea or "swansea's" or aberdeen or "aberdeen's" or dundee or "dundee's" or edinburgh or "edinburgh's" or glasgow or "glasgow's" or inverness or perth or "perth's" or stirling or "stirling's" or armagh or "armagh's" or belfast or "belfast's" or lisburn or "lisburn's" or londonderry or "londonderry's" or derry or "derry's" or newry or "newry's") | 7,845,725 |
| 3 | TS=((child* or infant* or newborn* or neonate* or postnatal or baby or babies or toddler*) and (immunis* or immuniz* or vaccin* or underimmunis* or underimmuniz* or undervaccinat* or DTaP or IPV or Hib or HepB or MenB or Rotavirus or PCV or MenC or MMR or LAIV or “Engerix B” or HBvaxPRO or “Infanrix hexa” or BCG)) | 78,138 |
| 2 | TS=(experience* or access* or view* or belief* or opinion* or assessment* or attitude* or feeling* or idea* or impression* or judgement* or "point of view*" or perspective* or perception* or concern* or accept* or hesita* or refus* or abstain* or declin* or resit* or object* or deny* or denier* or decision*) | 13,545,744 |
| 1 | TS=(parent* or mother* or father* or caregiver* or "care giver*" or stepfather* or stepmother*) | 967,585 |

**Social Policy and Practice**

| **#** | **Query** | **Results from 28 Jan 2022** |
| --- | --- | --- |
| 1 | (parent* or mother* or father* or caregiver* or "care giver*" or stepfather* or stepmother*).mp. | 43,788 |
| 2 | (experience* or access* or view* or belief* or opinion* or assessment* or attitude* or feeling* or idea* or impression* or judgement* or "point of view*" or perspective* or perception* or concern* or accept* or hesita* or refus* or abstain* or declin* or resit* or object* or deny* or denier* or decision*).mp. | 182,789 |
| 3 | ((child* or infant* or newborn* or neonate* or postnatal or baby or babies or toddler*) and (immunis* or immuniz* or vaccin* or underimmunis* or underimmuniz* or undervaccinat* or DTaP or IPV or Hib or HepB or MenB or Rotavirus or PCV or MenC or MMR or LAIV or Engerix B or HBvaxPRO or Infanrix hexa or BCG)).mp. | 541 |
| 4 | (national health service* or nhs*).mp. | 12,405 |
| 5 | english.mp. | 5,808 |
| 6 | (gb or "g.b." or britain* or british* or uk or "u.k." or united kingdom* or england* or northern ireland* or northern irish* or scotland* or scottish* or wales or welsh*).mp,jw. | 152,801 |
| 7 | (bath or "bath's" or birmingham or "birmingham's" or bradford or "bradford's" or brighton or "brighton's" or bristol or "bristol's" or carlisle* or "carlisle's" or cambridge or "cambridge's" or canterbury or "canterbury's" or chelmsford or "chelmsford's" or chester or "chester's" or chichester or "chichester's" or coventry or "coventry's" or derby or "derby's" or durham or "durham's" or ely or "ely's" or exeter or "exeter's" or gloucester or "gloucester's" or hereford or "hereford's" or hull or "hull's" or lancaster or "lancaster's" or leeds* or leicester or "leicester's" or lincoln or "lincoln's" or liverpool or "liverpool's" or london or "london's" or manchester or "manchester's" or newcastle or "newcastle's" or norwich or "norwich's" or nottingham or "nottingham's" or oxford or "oxford's" or peterborough or "peterborough's" or plymouth or "plymouth's" or portsmouth or "portsmouth's" or preston or "preston's" or ripon or "ripon's" or salford or "salford's" or salisbury or "salisbury's" or sheffield or "sheffield's" or southampton or "southampton's" or st albans or stoke or "stoke's" or sunderland or "sunderland's" or truro or "truro's" or wakefield or "wakefield's" or wells or westminster or "westminster's" or winchester or "winchester's" or wolverhampton or "wolverhampton's" or worcester or "worcester's" or york or "york's").mp. | 33,480 |
| 8 | (bangor or "bangor's" or cardiff or "cardiff's" or newport or "newport's" or st asaph or "st asaph's" or st davids or swansea or "swansea's").mp. | 771 |
| 9 | (aberdeen or "aberdeen's" or dundee or "dundee's" or edinburgh or "edinburgh's" or glasgow or "glasgow's" or inverness or perth or "perth's" or stirling or "stirling's").mp. | 4,412 |
| 10 | (armagh or "armagh's" or belfast or "belfast's" or lisburn or "lisburn's" or londonderry or "londonderry's" or derry or "derry's" or newry or "newry's").mp. | 467 |
| 11 | or/4-10 | 173,085 |
| 12 | 1 and 2 and 3 and 11 | 18 |
