# Supplementary material for: Parents’ and informal caregivers’ experiences of accessing childhood vaccination services within the United Kingdom: a systematic scoping review of empirical evidence
Source: BMC Public Health. 2024 Dec 18;24:3434. doi: 10.1186/s12889-024-20981-0 (PMC11653997; doi:10.1186/s12889-024-20981-0)
Supplement: Supplementary file 2 — Supplementary Material 2 [file 12889_2024_20981_MOESM2_ESM.docx]

**Additional File 2: Full-Text Exclusions List**

Articles identified through handsearching (i.e., screening of relevant reviews, reference searching, citation searching) are tabulated in grey.

**List of excluded articles: full-text screening (*n*=88)**

|  | **Reference** | **Reason for exclusion** |
| --- | --- | --- |
|  | Adams, J., McNaughton, R. J., Wigham, S., Flynn, D., Ternent, L., & Shucksmith, J. (2016). Acceptability of parental financial incentives and quasi-mandatory interventions for preschool vaccinations: triangulation of findings from three linked studies. *PloS one, 11*(6), 1-14. | Hypothetical/intention |
|  | Opel, D. J., Heritage, J., Taylor, J. A., Mangione-Smith, R., Salas, H. S., DeVere, V., ... & Robinson, J. D. (2013). The architecture of provider-parent vaccine discussions at health supervision visits. *Pediatrics, 132*(6), 1037-1046. | Not UK (US) |
|  | Bjornson, G. L., Scheifele, D. W., & Gold, R. (1997). Assessment of parent education methods for infant immunization. *Canadian journal of public health, 88*(6), 405-408. | Not UK (Canada) |
|  | Duffell, E. (2001). Attitudes of parents towards measles and immunisation after a measles outbreak in an anthroposophical community. *Journal of Epidemiology & Community Health, 55*(9), 685-686. | Vaccine hesitancy/ attitudes |
|  | Nowalk, M., & Lin, C. Beliefs and Attitudes about Influenza Immunization among Parents of Children with Chronic Medical Conditions over a Two-Year Period. *Journal of Urban Health: Bulletin of the New York Academy of Medicine, 83*(5), 1-10. | Not UK (US) |
|  | Canvin, K., Jones, C., Marttila, A., Burström, B., & Whitehead, M. (2007). Can I risk using public services? Perceived consequences of seeking help and health care among households living in poverty: qualitative study. *Journal of Epidemiology & Community Health, 61*(11), 984-989. | Not childhood immunisation (services generally) |
|  | Bhardwa, S. (2016). Childhood flu immunisation programme: Learning from the Scottish experience. *British Journal of School Nursing, 11*(6), 269-270. | Not parents/ caregivers |
|  | Fitzpatrick, P., Molloy, B., & Johnson, Z. (1997). Community mothers' programme: extension to the travelling community in Ireland. *Journal of Epidemiology & Community Health, 51*(3), 299-303. | Not UK (Republic of Ireland) |
|  | Johnson, Z., Howell, F., & Molloy, B. (1993). Community mothers' programme: randomised controlled trial of non-professional intervention in parenting. *British Medical Journal, 306*(6890), 1449-1452. | Not UK (Republic of Ireland) |
|  | Gorman, D. R., Bielecki, K., Larson, H. J., Willocks, L. J., Craig, J., & Pollock, K. G. (2020). Comparing vaccination hesitancy in Polish migrant parents who accept or refuse nasal flu vaccination for their children. *Vaccine, 38*(13), 2795-2799. | School-based programme |
|  | Tickner, S., Leman, P. J., & Woodcock, A. (2010). Design and validation of the Satisfaction With Immunisation Service Questionnaire (SWISQ). *Vaccine, 28*(36), 5883-5890. | Not childhood immunisation (methods) |
|  | Jones, A. E. (1984). Domiciliary immunisation for preschool child defaulters. *Br Med J (Clin Res Ed), 289*(6456), 1429-1431. | Not parents/ caregivers |
|  | Bjornson, G. L., Scheifele, D. W., LaJeunesse, C., & Bell, A. (1999). Effect of reminder notices on the timeliness of early childhood immunizations. *Paediatrics & Child Health, 4*(6), 400-405. | Not UK (Canada) |
|  | Emond, A., Pollock, J., Deave, T., Bonnell, S., Peters, T. J., & Harvey, I. (2002). An evaluation of the first parent health visitor scheme. *Archives of Disease in Childhood, 86*(3), 150-157. | Coverage/ epidemiological data |
|  | Stockwell, M. S., Irigoyen, M., Andres Martinez, R., & Findley, S. E. (2014). Failure to return: parental, practice, and social factors affecting missed immunization visits for urban children. *Clinical Pediatrics, 53*(5), 420-427. | Not UK (US) |
|  | Stevens, D., Baker, R., & Hands, S. (1986). Failure to vaccinate against whooping cough. *Archives of disease in childhood, 61*(4), 382-387. | Coverage/ epidemiological data |
|  | Hilton, S., Hunt, K., & Petticrew, M. (2007). Gaps in parental understandings and experiences of vaccine‐preventable diseases: a qualitative study. *Child: care, health and development, 33*(2), 170-179. | Vaccine hesitancy/ attitudes |
|  | Webb, E., Shankleman, J., Evans, M. R., & Brooks, R. (2001). The health of children in refuges for women victims of domestic violence: cross sectional descriptive survey. *BMJ, 323*(7306), 210-213. | Coverage/ epidemiological data |
|  | Brown, S., Hudson, D. B., Campbell-Grossman, C., & Yates, B. C. (2014). Health promotion text blasts for minority adolescent mothers. *MCN: The American Journal of Maternal/Child Nursing, 39*(6), 357-362. | Not UK (US) |
|  | Whiting, C. M., Schabas, R. E., & Ashley, M. J. (1990). Hib vaccine coverage in children attending day care/nursery school in East York. *Canadian Journal of Public Health= Revue Canadienne de Sante Publique, 81*(2), 152-155. | Not UK (Canada) |
|  | Saso, A., Skirrow, H., & Kampmann, B. (2020). Impact of COVID-19 on immunization services for maternal and infant vaccines: results of a survey conducted by imprint—the immunising pregnant women and infants network. *Vaccines, 8*(3), 556. | Not parents/ caregivers |
|  | Emery, D. P., Milne, T., Gilchrist, C. A., Gibbons, M. J., Robinson, E., Coster, G. D., ... & Grant, C. C. (2015). The impact of primary care on emergency department presentation and hospital admission with pneumonia: a case–control study of preschool-aged children. *NPJ primary care respiratory medicine, 25*(1), 1-10. | Not UK (New Zealand) |
|  | Athavale, D., McCullough, S., & Mactier, H. (2006). Implementing the new BCG vaccination guidelines—a maternity hospital-based clinic approach. *Journal of Public Health, 28*(2), 133-136. | Not parents/ caregivers |
|  | Jin, L., Téllez, P., Chia, R., Lu, D., Chadha, N. K., Pauwels, J., ... & Kozak, F. K. (2018). Improving vaccination uptake in pediatric Cochlear implant recipients. *Journal of Otolaryngology-Head & Neck Surgery, 47*(1), 1-7. | Not UK (Canada) |
|  | Clarke, S. J. (1980). Whooping cough vaccination: some reasons for non‐completion. *Journal of Advanced Nursing, 5*(3), 313-319. | Coverage/ epidemiological data |
|  | Casiday, R. (2006). Uncertainty, decision-making and trust: lessons from the MMR controversy. *Community Practitioner, 79*(11), 354. | Vaccine hesitancy/ attitudes |
|  | Feder, G. S, Vaclavik, T., Streetly, A. (1993). Traveller Gypsies and childhood immunization: a study in east London. *The British journal of general practice, 43*(372), 281. | Not parents/ caregivers |
|  | Stefanoff, P., Mamelund, S. E., Robinson, M., Netterlid, E., Tuells, J., Bergsaker, M. A. R., ... & VACSATC Working Group on Standardization of Attitudinal Studies in Europe. (2010). Tracking parental attitudes on vaccination across European countries: the Vaccine Safety, Attitudes, Training and Communication Project (VACSATC). *Vaccine, 28*(35), 5731-5737. | Vaccine hesitancy/ attitudes |
|  | Goomis, S. E. (2010). *Text messaging: An innovative educational method*. College of Saint Mary. | Not empirical article (book) |
|  | Hofstetter, A. M., DuRivage, N., Vargas, C. Y., Camargo, S., Vawdrey, D. K., Fisher, A., & Stockwell, M. S. (2015). Text message reminders for timely routine MMR vaccination: a randomized controlled trial. *Vaccine, 33*(43), 5741-5746. | Not UK (US) |
|  | Chan, C. (2000). A study of health services for the Chinese minority in Manchester. *British Journal of Community Nursing, 5*(3), 140-147. | Not childhood immunisation (services generally) |
|  | Hall, R., & Williams, A. L. (1988). Special advisory service for immunisation. *Archives of disease in childhood, 63*(12), 1498-1500. | Coverage/ epidemiological data |
|  | Stille, C. J., Christison-Lagay, J., Bernstein, B. A., & Dworkin, P. H. (2001). A simple provider-based educational intervention to boost infant immunization rates: a controlled trial. *Clinical pediatrics, 40*(7), 365-373. | Not UK (US) |
|  | Berry, N. J., Danchin, M., Trevena, L., Witteman, H. O., Kinnersley, P., Snelling, T., ... & Leask, J. (2018). Sharing knowledge about immunisation (SKAI): An exploration of parents’ communication needs to inform development of a clinical communication support intervention. *Vaccine, 36*(44), 6480-6490. | Not UK (Australia) |
|  | Casiday, R. E., & Cox, A. R. (2006). Restoring confidence in vaccines by explaining vaccine safety monitoring. *Drug safety, 29*(12), 1105-1109. | Not empirical article (commentary) |
|  | Fenick, A. M., Leventhal, J. M., Gilliam, W., & Rosenthal, M. S. (2020). A randomized controlled trial of group well-child care: Improved attendance and vaccination timeliness. *Clinical Pediatrics, 59*(7), 686-691. | Not UK (US) |
|  | Benin, A. L., Wisler-Scher, D. J., Colson, E., Shapiro, E. D., & Holmboe, E. S. (2006). Qualitative analysis of mothers' decision-making about vaccines for infants: the importance of trust. *Pediatrics, 117*(5), 1532-1541. | Not UK (US) |
|  | Armstrong, K. L., & Morris, J. (2000). Promoting secure attachment, maternal mood and child health in a vulnerable population: a randomized controlled trial. *Journal of paediatrics and child health, 36*(6), 555-562. | Not UK (Australia) |
|  | Murray, D. M. G. (2012). *A Pilot Study of Nurse Practitioner Facilitated Newborn Group Wellness Care*. University of Virginia. | Not empirical article (book) |
|  | Austin, H., Campion-Smith, C., Thomas, S., & Ward, W. (2008). Parents' difficulties with decisions about childhood immunization. *Community Practitioner, 81*(10), 32-36. | Vaccine hesitancy/ attitudes |
|  | Klein, N., Morgan, K., & Wansbrough-Jones, M. H. (1989). Parents' beliefs about vaccination: the continuing propagation of false contraindications. *BMJ: British Medical Journal, 298*(6689), 1687. | Coverage/ epidemiological data |
|  | Bennett, P., & Smith, C. (1992). Parents' attitudes towards immunisation in Wales according to socio-economic group: a preliminary investigation. *Health Education Journal, 51*(3), 127-131. | Vaccine hesitancy/ attitudes |
|  | Flynn, D., Ternent, L., Becker, F., Oluboyede, Y., & Adams, J. (2017). Parental preferences for the Organization of Preschool Vaccination Programs Including Financial Incentives: a discrete choice experiment. *MDM Policy & Practice, 2*(1), 1-13. | Hypothetical/intention |
|  | Humiston, S. G., Lerner, E. B., Hepworth, E., Blythe, T., & Goepp, J. G. (2005). Parent opinions about universal influenza vaccination for infants and toddlers. *Archives of pediatrics & adolescent medicine, 159*(2), 108-112. | Not UK (US) |
|  | Plumptre, I., Tolppa, T., & Blair, M. (2020). Parent and staff attitudes towards in-hospital opportunistic vaccination. *Public Health, 182*, 39-44. | Catch-up campaign |
|  | Poltorak, M., Leach, M., Fairhead, J., & Cassell, J. (2005). ‘MMR talk’and vaccination choices: An ethnographic study in Brighton. *Social Science & Medicine, 61*(3), 709-719. | Vaccine hesitancy/ attitudes |
|  | Sadler, L. S., Slade, A., Close, N., Webb, D. L., Simpson, T., Fennie, K., & Mayes, L. C. (2013). Minding the baby: Enhancing reflectiveness to improve early health and relationship outcomes in an interdisciplinary home‐visiting program. *Infant mental health journal, 34*(5), 391-405. | Not UK (US) |
|  | Blair, S., Shave, N., & McKay, J. (1985). Measles matters, but do parents know?. *Br Med J (Clin Res Ed), 290*(6468), 623-624. | Vaccine hesitancy/ attitudes |
|  | Harrington, P. M., Woodman, C., & Shannon, W. F. (2000). Low immunisation uptake: Is the process the problem?. *Journal of Epidemiology & Community Health, 54*(5), 394-394. | Not UK (Republic of Ireland) |
|  | Morgan, M. Z., & Evans, M. R. (1998). Initiatives to improve childhood immunisation uptake: a randomised controlled trial. *BMJ, 316*(7144), 1570-1571. | Coverage/ epidemiological data |
|  | Falconer, M. A. (2013). Improving vaccine uptake: an overview. *Human vaccines & immunotherapeutics, 9*(6), 1368-1371. | Catch-up campaign |
|  | Brambleby, P., & Hanrahan, J. (1989). Measles immunisation non-acceptance: validation of computer-held records and raising the vaccine uptake at early school age; the Maidstone experience. *Public Health, 103*(4), 289-294. | Coverage/ epidemiological data |
|  | Sutton, G. C. (1991). Mumps, measles and rubella vaccination: a pragmatic study. *Public Health, 105*(2), 133-138. | Biological |
|  | O’Brien, N., Joyce, B., Hayes, A. M., Bedford, H., & Quinn, N. (2021). Parental perceptions regarding the impact of housing on health. *Archives of Disease in Childhood,106*, 250 | Not empirical article (conference abstract) |
|  | Daniels, J. (2002). Parents found it difficult to obtain objective information and advice about the combined mumps, measles, and rubella vaccine. *Evidence-based nursing, 5*(4), 122. | Vaccine hesitancy/ attitudes |
|  | Cheater, F. M. (2006). Mothers' decisions about MMR vaccination were framed by their children's vulnerabilities and wider social trends. *Evidence-Based Nursing, 9*(1), 27-27. | Not empirical article (commentary) |
|  | MacDonald, M. (2005). Parents' decisions on MMR vaccination for their children were based on personal experience rather than scientific evidence. *Evidence-based nursing, 8*(2), 60. | Not empirical article (commentary) |
|  | Isba, R., Cleland, J., Cooke, D., Lees, A., Higgins, S., & Hilditch, K. (2017). Pilot of opportunistic seasonal influenza vaccination for children attending the paediatric emergency department. *Archives of Disease in Childhood, 102*, 109-110. | Not empirical article (conference abstract) |
|  | Myttaraki, E., Soni, A., & Watson, M. (2021). A sample survey: parental views on routine childhood vaccination, the flu and COVID vaccines during the pandemic. *Archives of Disease in Childhood, 106*, 184. | Not empirical article (conference abstract) |
|  | Nelson, K. G., Key, D., Fletcher, J. K., Kirkpatrick, E., & Feinstein, R. (1982). The teen—Tot clinic: An alternative to traditional care for infants of teenaged mothers. *Journal of Adolescent Health Care, 3*(1), 19-23. | Not UK (US) |
|  | Devanahalli, C., Kapoor, S., Burnside, D., & Radcliffe, R. (2020). Vacs: vaccination awareness in children study–a study to look into perceptions and assumptions among parents and clinicians. *Archives of Disease in Childhood, 105*, 165. | Not empirical article (conference abstract) |
|  | Bhrolchain, C. N. (1994). Will deprived areas still need community paediatricians? An unpopular hypothesis. Pub*lic health, 108*(5), 319-326. | Coverage/ epidemiological data |
|  | Plumptre, I., Tolppa, T., & Blair, M. (2018). An audit of vaccination adherence and acceptability of opportunistic vaccination in inpatient paediatrics. *Archives of Disease in Childhood, 103*, 176 | Not empirical article (conference abstract) |
|  | Finlay, F., McKechnie, L., Pearce, A., & Lenton, S. (1999). Immunization telephone hotline audit Commentary by Gary L Freed. *Ambulatory child health, 5*(4), 295-302. | Coverage/ epidemiological data |
|  | Currie, A. L., Gehlbach, S. H., Massion, C., & Thompson, S. (1983). Newborn home visits. *J Fam Pract, 17*(4), 635-638. | Coverage/ epidemiological data |
|  | London School of Hygiene and Tropical Medicine. (1964). The knowledge and attitudes of mothers to immunization. *Medical Officer, 112* (17), 231-235. | Coverage/ epidemiological data |
|  | Riley, D. J., Mughal, M. Z., & Roland, J. (1991). Immunisation state of young children admitted to hospital and effectiveness of a ward based opportunistic immunisation policy. *British medical journal, 302*(6767), 31-33. | Catch-up campaign |
|  | Hopper, C., & Chedeville, G. (2020). Influenza Immunization: A Quality Assurance Survey in a Pediatric Rheumatology Clinic. *In Arthritis & Rheumatology, 72*, 315-316. | Not empirical article (conference abstract) |
|  | Eales, M., Wilkinson, A., Hall, G. W., Bhatnagar, N., & Pool, S. (2021). A review following implemented training for health care practitioners in primary care settings of immunisation practice in children with bleeding disorders. Res*earch and Practice in Thrombosis and Haemostasis, 5*. | Not empirical article (conference abstract) |
|  | Cullen, J. (2005). Why parents choose not to vaccinate their children against childhood diseases. *Professional Nurse, 20*(5), 31-33. | Not empirical article (magazine article) |
|  | Hampshire, A. J., Blair, M. E., Crown, N. S., Avery, A. J., & Williams, E. I. (2002). Assessing the quality of preschool child health surveillance in primary care: a pilot study in one health district. *Child: care, health and development, 28*(3), 239-249. | Not childhood immunisation (services generally) |
|  | Fisher-Jeffes, L., & Finlay, F. (2006). Immunisation hotline calls as five-in-one vaccine introduced. *Community Practitioner, 79*(4), 121-124. | Not parents/caregivers |
|  | Tickner, S., Leman, P. J., & Woodcock, A. (2007). ‘It's just the normal thing to do’: exploring parental decision-making about the ‘five-in-one’vaccine. *Vaccine, 25*(42), 7399-7409. | Hypothetical/intention |
|  | Shourie, S., Jackson, C., Cheater, F. M., Bekker, H. L., Edlin, R., Tubeuf, S., ... & Hammond, L. (2013). A cluster randomised controlled trial of a web based decision aid to support parents’ decisions about their child's Measles Mumps and Rubella (MMR) vaccination. *Vaccine, 31*(50), 6003-6010. | Hypothetical/intention |
|  | Jackson, C., Cheater, F. M., Peacock, R., Leask, J., & Trevena, L. (2010). Evaluating a web-based MMR decision aid to support informed decision-making by UK parents: A before-and-after feasibility study. *Health Education Journal, 69*(1), 74-83. | Hypothetical/intention |
|  | Yarnell, J. (1976). Evaluation of health education: The use of a model of preventive health behaviour. *Social Science & Medicine, 10*(7-8), 393-398. | Catch-up campaign |
|  | Petrovic, M., Roberts, R. J., Ramsay, M., & Charlett, A. (2003). Parents' attitude towards the second dose of measles, mumps and rubella vaccine: a case-control study. *Communicable disease and public health, 6*(4), 325-329. | Unable to retrieve |
|  | Abdulrahman, G. O. (2012). Child healthcare in the United Kingdom and Nigeria. *Ethiopian Medical Journal, 50*(1), 99-101. | Unable to retrieve |
|  | Wroe, A. L., Turner, N., & Owens, R. G. (2005). Evaluation of a decision-making aid for parents regarding childhood immunizations. *Health Psychology, 24*(6), 539. | Unable to retrieve |
|  | Tung, S., Pearce, A., & Bedford, H. (2012). Factors determining uptake of the pre-school booster vaccine: findings from the UK Millennium Cohort Study. *Archives of Disease in Childhood, 97*(1), 64-65. | Unable to retrieve |
|  | Thomson, W. O. (1959). Why do parents refuse immunization? *Medical Press, 241*(10), 217-219. | Unable to retrieve |
|  | Tellez, P., Pauwels, J., Jin, C., Lu, D., Dobson, S., & Kozak, F. K. (2016). Assessment and comparison of vaccination status in pediatric cochlear implant patients: A 10 year retrospective review. *Journal of Investigative Medicine*, 64(1), 322-323. | Unable to retrieve |
|  | Davies, M., Logan, J., & Carter, H. (1995). Monitoring the uptake of Hib vaccine in Forth Valley. P*rofessional Care of Mother and Child, 5*(6), 168-170. | Unable to retrieve |
|  | Rowland, A. J. (1965). Motives of Bradford Mothers in accepting or rejecting Smallpox Vaccination for their Children. *Medical Officer, 114*(22), 289-95. | Unable to retrieve |
|  | Baxter, D. (1995). The organization, delivery and audit of a specialist immunization clinic. *Journal of Management in Medicine, 9*(1), 58-65. | Unable to retrieve |
|  | Ko, M. L., Rao, M., Teare, L., Bridgman, G. C., & Kurian, A. (1995). Outcome of referrals to a district immunisation advisory clinic. Communicable Disease report. *CDR Review, 5*(10), 146-9. | Unable to retrieve |
|  | Sharp, J. C. M. (1962). Parental Attitudes to Smallpox Vaccination. *Medical Officer, 107*(13), 193-5. | Unable to retrieve |
|  | Eales, M., Wilkinson, A., Pool, S., Hall, G. W., & Bhatnagar, N. (2019). A single centre patient/parent experience of immunisation practice in children with bleeding disorders. *Haemophilia, 25*, 135. | Unable to retrieve |
|  | Dalphinis, J. (1986). Do immunisation defaulters know enough about immunisation?. *Health visitor, 59*(11), 342-344. | Unable to retrieve |
|  | Bedford, H. (1990). Achieving immunisation targets: the health visitor's role. *Health Visitor, 63*(1990), 416-418. | Unable to retrieve |
|  | Mason, B. W., & Donnelly, P. D. (2000). Targeted mailing of information to improve uptake of measles, mumps, and rubella vaccine: a randomised controlled trial. *Communicable Disease and Public Health, 3*(1), 67-68. | Unable to retrieve |

**List of included articles: full-text screening (*n*=46)**

|  | **Reference** | **Intervention (Y/N)** |
| --- | --- | --- |
|  | Gardner, B., Davies, A., McAteer, J., & Michie, S. (2010). Beliefs underlying UK parents' views towards MMR promotion interventions: a qualitative study. *Psychology, health & medicine, 15*(2), 220-230. | N |
|  | Sporton, R. K., & Francis, S. A. (2001). Choosing not to immunize: are parents making informed decisions?. *Family Practice, 18*(2), 181-188. | N |
|  | Condon, L., McClean, S., & McRae, L. (2020). ‘Differences between the earth and the sky’: migrant parents’ experiences of child health services for pre-school children in the UK. *Primary Health Care Research & Development, 21*(e29), 1-8. | N |
|  | Mixer, R. E., Jamrozik, K., & Newsom, D. (2007). Ethnicity as a correlate of the uptake of the first dose of mumps, measles and rubella vaccine. *Journal of Epidemiology & Community Health, 61*(9), 797-801. | N |
|  | Lakhani, A. D., Avery, A., Gordon, A., & Tait, N. (1984). Evaluation of a home based health record booklet. *Archives of Disease in Childhood, 59*(11), 1076-1081. | Y (communication/ information) |
|  | Atchison, C., Zvoc, M., & Balakrishnan, R. (2013). The evaluation of a standardized call/recall system for childhood immunizations in Wandsworth, England. *Journal of community health, 38*(3), 581-587. | Y (communication/ information) |
|  | Newton, P., & Smith, D. M. (2017). Factors influencing uptake of measles, mumps and rubella (MMR) immunization in site‐dwelling Gypsy, Roma and Traveller (G&T) communities: a qualitative study of G&T parents' beliefs and experiences. *Child: care, health and development, 43*(4), 504-510. | N |
|  | Tomlinson, N., & Redwood, S. (2013). Health beliefs about preschool immunisations: an exploration of the views of Somali women resident in the UK. *Diversity & Equality in Health and Care, 10*(2), 101-113. | N |
|  | Petts, J., & Niemeyer, S. (2004). Health risk communication and amplification: learning from the MMR vaccination controversy. *Health, risk & society, 6*(1), 7-23. | N |
|  | Bell, S., Edelstein, M., Zatoński, M., Ramsay, M., & Mounier-Jack, S. (2019). ‘I don’t think anybody explained to me how it works’: qualitative study exploring vaccination and primary health service access and uptake amongst Polish and Romanian communities in England. *BMJ open, 9*(7), 1-9. | N |
|  | Guillaume, L. R., & Bath, P. A. (2004). The impact of health scares on parents’ information needs and preferred information sources: a case study of the MMR vaccine scare. *Health Informatics Journal, 10*(1), 5-22. | N |
|  | Ellis, N., Walker-Todd, E., & Heffernan, C. (2020). Influences on childhood immunisation decision-making in London's Gypsy and Traveller communities. *British Journal of Nursing, 29*(14), 822-826. | N |
|  | Carter, H., & Jones, I. G. (1985). Measles immunisation: results of a local programme to increase vaccine uptake. *Br Med J (Clin Res Ed), 290*(6483), 1717-1719. | Y (communication/ information) |
|  | Smailbegovic, M. S., Laing, G. J., & Bedford, H. (2003). Why do parents decide against immunization? The effect of health beliefs and health professionals. *Child: care, health and development, 29*(4), 303-311. | N |
|  | Lewendon, G. J., & Maconachie, M. (2002). Why are children not being immunised? Barriers to immunisation uptake in South Devon. *Health education journal,* 61(3), 212-220. | N |
|  | Jackson C, Dyson L, Bedford H, Cheater FM, Condon L, Crocker A, et al. (2016). UNderstanding uptake of Immunisations in TravellIng aNd Gypsy communities (UNITING): a qualitative interview study. *Health Technol Assess*, 20(72), 1-208. | N |
|  | Yarwood, J., Noakes, K., Kennedy, D., Campbell, H., & Salisbury, D. (2005). Tracking mothers attitudes to childhood immunisation 1991–2001. *Vaccine, 23*(48-49), 5670-5687. | N |
|  | Johnson, S., & Capdevila, R. (2014). ‘That’s just what’s expected of you… so you do it’: Mothers discussions around choice and the MMR vaccination. *Psychology & health, 29*(8), 861-876. | N |
|  | Letley, L., Rew, V., Ahmed, R., Habersaat, K. B., Paterson, P., Chantler, T., ... & Butler, R. (2018). Tailoring immunisation programmes: using behavioural insights to identify barriers and enablers to childhood immunisations in a Jewish community in London, UK. *Vaccine, 36*(31), 4687-4692. | N |
|  | Smith, D., & Newton, P. (2017). Structural barriers to measles, mumps and rubella (MMR) immunisation uptake in Gypsy, Roma and Traveller communities in the United Kingdom. *Critical Public Health, 27*(2), 238-247. | N |
|  | McHale, P., Keenan, A., & Ghebrehewet, S. (2016). Reasons for measles cases not being vaccinated with MMR: investigation into parents' and carers' views following a large measles outbreak. *Epidemiology & Infection, 144*(4), 870-875. | N |
|  | Lwembe, S., Green, S. A., Tanna, N., Connor, J., Valler, C., & Barnes, R. (2016). A qualitative evaluation to explore the suitability, feasibility and acceptability of using a ‘celebration card’ intervention in primary care to improve the uptake of childhood vaccinations. *BMC family practice, 17*(1), 1-11. | Y (communication/ information) |
|  | Henderson, L., Millett, C., & Thorogood, N. (2008). Perceptions of childhood immunization in a minority community: qualitative study. *Journal of the Royal Society of Medicine, 101*(5), 244-251. | N |
|  | Tickner, S., Leman, P. J., & Woodcock, A. (2010). Parents' views about pre‐school immunization: an interview study in southern England. *Child: care, health and development, 36*(2), 190-197. | N |
|  | Bell, S., Clarke, R., Paterson, P., & Mounier-Jack, S. (2020). Parents’ and guardians’ views and experiences of accessing routine childhood vaccinations during the coronavirus (COVID-19) pandemic: A mixed methods study in England. *PloS one, 15*(12), e0244049. | N |
|  | Lunts, E., & Cowper, D. (2002). Parents refusing MMR: do GPs and health visitors understand why?. *Community practitioner, 75*(3), 94. | N |
|  | Bennett, P., & Smith, C. (1992). Parents attitudinal and social influences on childhood vaccination. *Health education research, 7*(3), 341-348. | N |
|  | Sampson, R., Wong, L., & MacVicar, R. (2011). Parental reasons for non-uptake of influenza vaccination in young at-risk groups: a qualitative study. *British Journal of General Practice, 61*(588), 1-6. | N |
|  | Conway, S. P. (1999). Opportunistic immunisation in hospital. *Archives of disease in childhood, 81*(5), 422-425. | Y (opportunistic vaccination: location, finances, organisation, communication) |
|  | Lakhani, A. D., Morris, R. W., Morgan, M., Dale, C., & Vaile, M. S. (1987). Measles immunisation: feasibility of a 90% target uptake. *Archives of disease in childhood, 62*(12), 1209-1214. | N |
|  | Condon, L. (2002). Maternal attitudes to preschool immunisations among ethnic minority groups. *Health Education Journal, 61*(2), 180-189. | N |
|  | McMurray, R., Cheater, F. M., Weighall, A., Nelson, C., Schweiger, M., & Mukherjee, S. (2004). Managing controversy through consultation: a qualitative study of communication and trust around MMR vaccination decisions. *British Journal of General Practice, 54*(504), 520-525. | N |
|  | Morgan, S., Aslam, M., Dove, R., Nicoll, A., & Stanford, R. (1987). Knowledge of infectious diseases and immunisation among Asian and white parents. *Health Education Journal, 46*(4), 177-179. | N |
|  | Bedford, H., & Lansley, M. (2006). Information on childhood immunisation: parents' views. *Community Practitioner, 79*(8), 252-255. | N |
|  | Hill, M. C., & Cox, C. L. (2013). Influencing factors in MMR immunisation decision making. *British Journal of Nursing, 22*(15), 893-898. | N |
|  | Austin, H. (2001). Parents' perceptions of information on immunisations. *Journal of Child Health Care: for Professionals Working with Children in the Hospital and Community, 5*(2), 54-59. | N |
|  | New, S. J., & Senior, M. L. (1991). “I don't believe in needles”: Qualitative aspects of a study into the uptake of infant immunisation in two english health authorities. *Social Science & Medicine, 33*(4), 509-518. | N |
|  | Cuninghame, C. J., Charlton, C. P. J., & Jenkins, S. M. (1994). Immunization uptake and parental perceptions in a strictly orthodox Jewish community in north-east London. *Journal of Public Health, 16*(3), 314-317. | N |
|  | Adjaye, N. (1981). Measles immunization. Some factors affecting non-acceptance of vaccine. *Public Health, 95*(4), 185-188. | N |
|  | Newton, S. (2006). Parental acceptability of a pneumococcal conjugate vaccine in the UK childhood immunisation schedule. *Primary Health Care, 16*(8), 34. | N |
|  | Gorman, D. R., Bielecki, K., Willocks, L. J., & Pollock, K. G. (2019). A qualitative study of vaccination behaviour amongst female Polish migrants in Edinburgh, Scotland. *Vaccine, 37*(20), 2741-2747. | N |
|  | Jackson, C., Cheater, F. M., Harrison, W., Peacock, R., Bekker, H., West, R., & Leese, B. (2011). Randomised cluster trial to support informed parental decision-making for the MMR vaccine. *BMC public health, 11*(1), 1-11. | Y (communication/ information) |
|  | SQW, Ipsos MORI, Karl Ashworth, Bristol University, & CRG Consulting. (2011). *Evaluation of Flying Start*. Welsh Government. https://gov.wales/sites/default/files/statistics-and-research/2019-04/evaluation-of-flying-start-findings-from-the-baseline-survey-of-families.pdf | Y (Location  Supply/demand  Financial  Organisation  Communication/ information) |
|  | Jackson, C., Bedford, H., Cheater, F. M., Condon, L., Emslie, C., Ireland, L., ... & Dyson, L. (2017). *Needles, Jabs and Jags: a qualitative exploration of barriers and facilitators to child and adult immunisation uptake among Gypsies, Travellers and Roma*. BMC Public Health, 17(1), 1-17. | N |
|  | Morgan, M., Lakhani, A. D., Morris, R. W., Dale, C., & Vaile, M. S. B. (1987b). Parents' attitudes to measles immunization. *The Journal of the Royal College of General Practitioners, 37*(294), 25-27. | N |
|  | Bell, S., Saliba, V., Ramsay, M., & Mounier-Jack, S. (2020). What have we learnt from measles outbreaks in 3 English cities? A qualitative exploration of factors influencing vaccination uptake in Romanian and Roma Romanian communities. *BMC public health, 20*(1), 1-10. | N |

**Summary of related reviews screened for relevant primary articles - identified from database searches (*n*=33)**

|  | **Reference** |
| --- | --- |
|  | Forster, A. S., Rockliffe, L., Chorley, A. J., Marlow, L. A., Bedford, H., Smith, S. G., & Waller, J. (2017). Ethnicity-specific factors influencing childhood immunisation decisions among Black and Asian Minority Ethnic groups in the UK: a systematic review of qualitative research. *J Epidemiol Community Health, 71*(6), 544-549. |
|  | Roberts, K. A., Dixon-Woods, M., Fitzpatrick, R., Abrams, K. R., & Jones, D. R. (2002). Factors affecting uptake of childhood immunisation: a Bayesian synthesis of qualitative and quantitative evidence. *The Lancet, 360*(9345), 1596-1599. |
|  | de Cantuária Tauil, M., Sato, A. P. S., & Waldman, E. A. (2016). Factors associated with incomplete or delayed vaccination across countries: a systematic review. *Vaccine, 34*(24), 2635-2643. |
|  | Falagas, M. E., & Zarkadoulia, E. (2008). Factors associated with suboptimal compliance to vaccinations in children in developed countries: a systematic review. *Current medical research and opinion, 24*(6), 1719-1741. |
|  | Tickner, S., Leman, P. J., & Woodcock, A. (2006). Factors underlying suboptimal childhood immunisation. *Vaccine, 24*(49-50), 7030-7036. |
|  | Lochhead, Y. J. (1991). Failure to immunize children under 5 years: a literature review. *Journal of Advanced Nursing, 16*(2), 130-137. |
|  | Norman, D. A., Barnes, R., Pavlos, R., Bhuiyan, M., Alene, K. A., Danchin, M., ... & Blyth, C. C. (2021). Improving influenza vaccination in children with comorbidities: A systematic review. *Pediatrics, 147*(3). |
|  | Kurup, L., Shorey, S., Wang, W., & He, H. G. (2017). An integrative review on parents’ perceptions of their children’s vaccinations. *Journal of Child Health Care, 21*(3), 343-352. |
|  | Lewin, S., Munabi‐Babigumira, S., Glenton, C., Daniels, K., Bosch‐Capblanch, X., Van Wyk, B. E., ... & Scheel, I. B. (2010). Lay health workers in primary and community health care for maternal and child health and the management of infectious diseases. *Cochrane database of systematic reviews*, (3). |
|  | Whittaker, K. (2002). Lay workers for improving the uptake of childhood immunization. Br*itish journal of community nursing, 7*(9), 474-479. |
|  | Kaufman, J., Tuckerman, J., Bonner, C., Durrheim, D. N., Costa, D., Trevena, L., ... & Danchin, M. (2021). Parent-level barriers to uptake of childhood vaccination: a global overview of systematic reviews. *BMJ global health, 6*(9), e006860. |
|  | Ames, H. M., Glenton, C., & Lewin, S. (2017). Parents' and informal caregivers' views and experiences of communication about routine childhood vaccination: a synthesis of qualitative evidence. *Cochrane Database of Systematic Reviews*, (2). |
|  | Williams, N., Woodward, H., Majeed, A., & Saxena, S. (2011). Primary care strategies to improve childhood immunisation uptake in developed countries: systematic review. *JRSM short reports, 2*(10), 1-21. |
|  | Yemeke, T. T., Mitgang, E., Wedlock, P. T., Higgins, C., Chen, H. H., Pallas, S. W., ... & Ozawa, S. (2021). Promoting, seeking, and reaching vaccination services: A systematic review of costs to immunization programs, beneficiaries, and caregivers. *Vaccine, 39*(32), 4437-4449. |
|  | Connors, J. T., Slotwinski, K. L., & Hodges, E. A. (2017). Provider-parent communication when discussing vaccines: a systematic review. *Journal of pediatric nursing, 33*, 10-15. |
|  | Wilder-Smith, A. B., & Qureshi, K. (2020). Resurgence of measles in Europe: a systematic review on parental attitudes and beliefs of measles vaccine. *Journal of epidemiology and global health, 10*(1), 46. |
|  | Carandang, R. R., Sakamoto, J. L., Kunieda, M. K., Shibanuma, A., Yarotskaya, E., Basargina, M., & Jimba, M. (2021). Roles of the maternal and child health handbook and other home-based records on newborn and child health: A systematic review. *International journal of environmental research and public health, 18*(14), 7463. |
|  | Bocquier, A., Ward, J., Raude, J., Peretti-Watel, P., & Verger, P. (2017). Socioeconomic differences in childhood vaccination in developed countries: a systematic review of quantitative studies. *Expert review of vaccines, 16*(11), 1107-1118. |
|  | Smith, L. E., Amlôt, R., Weinman, J., Yiend, J., & Rubin, G. J. (2017). A systematic review of factors affecting vaccine uptake in young children. *Vaccine, 35*(45), 6059-6069. |
|  | Mills, E., Jadad, A. R., Ross, C., & Wilson, K. (2005). Systematic review of qualitative studies exploring parental beliefs and attitudes toward childhood vaccination identifies common barriers to vaccination. *Journal of clinical epidemiology, 58*(11), 1081-1088. |
|  | Stewart, J., & Sayer, L. (2021). What factors influence measles, mumps and rubella vaccine hesitancy among parents? A systematic review. *British Journal of Child Health, 2*(3), 143-152. |
|  | de Cock, C., van Velthoven, M., Milne-Ives, M., Mooney, M., & Meinert, E. (2020). Use of apps to promote childhood vaccination: systematic review. *JMIR mHealth and uHealth, 8*(5), e17371. |
|  | Corben, P., & Leask, J. (2016). To close the childhood immunization gap, we need a richer understanding of parents' decision-making. *Human Vaccines & Immunotherapeutics, 12*(12), 3168-3176. |
|  | Palmer, M. J., Henschke, N., Bergman, H., Villanueva, G., Maayan, N., Tamrat, T., ... & Free, C. (2020). Targeted client communication via mobile devices for improving maternal, neonatal, and child health. C*ochrane Database of Systematic Reviews,* (8). |
|  | Haroune, V., & King, L. (2020). Factors contributing to parental ‘vaccine hesitancy’ for childhood immunisations. *Nursing children and young people, 32*(4). |
|  | Crescitelli, M. D., Ghirotto, L., Sisson, H., Sarli, L., Artioli, G., Bassi, M. C., ... & Hayter, M. (2020). A meta-synthesis study of the key elements involved in childhood vaccine hesitancy. *Public Health, 180*, 38-45. |
|  | Mihalek, A. J., Kysh, L., & Pannaraj, P. S. (2019). Pediatric inpatient immunizations: a literature review. *Hospital Pediatrics, 9*(7), 550-559. |
|  | Kempe, A., Stockwell, M. S., & Szilagyi, P. (2021). The contribution of reminder-recall to vaccine delivery efforts: a narrative review. *Academic Pediatrics, 21*(4), S17-S23. |
|  | Frew, P. M., & Lutz, C. S. (2017). Interventions to increase pediatric vaccine uptake: An overview of recent findings. *Human vaccines & immunotherapeutics, 13*(11), 2503-2511. |
|  | Torracinta, L., Tanner, R., & Vanderslott, S. (2021). Mmr vaccine attitude and uptake research in the united kingdom: a critical review. *Vaccines, 9*(4), 402. |
|  | Forster, A. S., Rockliffe, L., Chorley, A. J., Marlow, L. A., Bedford, H., Smith, S. G., & Waller, J. (2016). A qualitative systematic review of factors influencing parents’ vaccination decision-making in the United Kingdom. *SSM-population health*, 2, 603-612. |
|  | Hussin, H., & Marzo, R. R. (2020). A literature review of parental barriers to child immunizations. *J Crit Rev, 7*(3), 642-6. |

**Summary of related reviews screened for relevant primary articles – known to the authors (*n*=1)**

|  | **Reference** |
| --- | --- |
|  | Cooper, S., Schmidt, B. M., Sambala, E. Z., Swartz, A., Colvin, C. J., Leon, N., & Wiysonge, C. S. (2021). Factors that influence parents' and informal caregivers' views and practices regarding routine childhood vaccination: a qualitative evidence synthesis. *Cochrane Database of Systematic Reviews,* (10). |
