# Supplementary material for: Parents’ and informal caregivers’ experiences of accessing childhood vaccination services within the United Kingdom: a systematic scoping review of empirical evidence
Source: BMC Public Health. 2024 Dec 18;24:3434. doi: 10.1186/s12889-024-20981-0 (PMC11653997; doi:10.1186/s12889-024-20981-0)
Supplement: Supplementary file 3 — Supplementary Material 3 [file 12889_2024_20981_MOESM3_ESM.docx]

**Additional File 3: Data Extraction Instrument (Implemented in Excel)**

Tab 1: Not intervention

| Lead author | Year | Aims/ purpose of study | Vaccine(s) considered | Definition of accessibility | Accessibility factors reported (definition) | | | | | | | Views/experiences reported | Method/study design | | | Accessibility recommendations reported | | | | | | | Recommendations for research | Partial/full data | Notes |
| --- | --- | --- | --- | --- | --- | --- | --- | --- | --- | --- | --- | --- | --- | --- | --- | --- | --- | --- | --- | --- | --- | --- | --- | --- | --- |
|  |  |  |  |  | *1* | *2* | *3* | *4* | *5* | *6* | *Other* |  | *Theories/ models used* | *Study design* | *Population(s)/ group(s) studied (setting)* | *1* | *2* | *3* | *4* | *5* | *6* | *Other* |  |  |  |

Tab 2: Intervention

| Lead author | Year | Intervention type | Intervention [development] | *Population(s)/ group(s) studied (setting)* | Vaccine(s) considers | *Theories/ models used* | *Study design* | Result | Definition of accessibility | Accessibility factors reported (definition) | | | | | | | Views/experiences reported | Accessibility recommendations reported | | | | | | | Recommendations for research | Partial/ full data | Notes |
| --- | --- | --- | --- | --- | --- | --- | --- | --- | --- | --- | --- | --- | --- | --- | --- | --- | --- | --- | --- | --- | --- | --- | --- | --- | --- | --- | --- |
|  |  |  |  |  |  |  |  |  |  | *1* | *2* | *3* | *4* | *5* | *6* | *Other* |  | *1* | *2* | *3* | *4* | *5* | *6* | *Other* |  |  |  |
