# Supplementary material for: Parents’ and informal caregivers’ experiences of accessing childhood vaccination services within the United Kingdom: a systematic scoping review of empirical evidence
Source: BMC Public Health. 2024 Dec 18;24:3434. doi: 10.1186/s12889-024-20981-0 (PMC11653997; doi:10.1186/s12889-024-20981-0)
Supplement: Supplementary file 4 — Supplementary Material 4 [file 12889_2024_20981_MOESM4_ESM.docx]

**Additional File 4: Characteristics of Included Studies**

**Non-intervention studies**

| **Author (year)** | **Aim(s)/objective(s) of study** | **Participants (n) [setting]** | **Vaccines considered** | **Guiding theory/ model** | **Method (study design)** | **Partial/ Full data extraction** |
| --- | --- | --- | --- | --- | --- | --- |
| 1. Gardner (2010) | To explore beliefs towards MMR vaccination, including potential motivational and organisational interventions to boost MMR vaccination | Parents (n=28)  [5 Primary Care Trust areas with uptake below recommended (95%) coverage: Greenwich, Westminster, Sutton and Merton, Brent, and Camden] | MMR | N/a | Qualitative (focus groups) | P |
| 1. Sporton (2001) | To explore the decision-making process of parents who have chosen not to have their children immunised | Parents (n=13)  [Unspecified ‘inner city centre’ with lower than national average immunisation coverage] | Childhood immunisation generally | N/a | Qualitative (semi-structured interviews) | P |
| 1. Condon (2020) | To explore parents’ experiences of using child health services for their pre-school children post-migration | Migrants from Romania, Poland, Pakistan or Somalia (n=28)  [South West England] | Childhood immunisation generally | N/a | Qualitative (focus groups) | P |
| 1. Mixer (2007) | To investigate whether a relationship exists between ethnicity and uptake of the first dose of MMR vaccination, and to study important factors influencing the parental decision about vaccination | Mothers from Asian, Afro-Caribbean and White backgrounds (n=37)  [Brent, North-west London] | MMR | N/a | Qualitative ([1] focus groups, [2] questionnaire: same sample) | P |
| 1. Newton (2017) | To explore Gypsy, Roma and Traveller (G&T) parents: (1) beliefs about childhood immunization; (2) beliefs about the risks of immunization and non- immunization; (3) perceived obstacles to, and facilitators of, immunization and (4) views on increasing immunization levels. | Site-dwelling G&T women with pre-school aged children (n=16)  [Kent in South-East England, sites were chosen which had recently experienced measles outbreaks] | MMR | N/a | Qualitative (focus groups) | P |
| 1. Tomlinson (2013) | To explore the views of the Somali population in the UK with regard to preschool immunisation as a basis for ensuring a culturally appropriate service | Born in Somalia, the mother of at least one child under 5 years old (n=23)  [Birmingham] | Childhood immunisation generally | N/a | Qualitative (semi-structured interviews) | P |
| 1. Petts (2004) | To explore the impacts of experience, mediated knowledge and social context upon information needs and understanding. | Parents (n=64)  [West Midlands area, centred on Birmingham and Nuneaton, take-up rates consistent with the national average] | MMR | N/a | Qualitative ([1] focus group, [2] focus group: same sample) | P |
| 1. Bell (2019) | To explore vaccination attitudes and behaviours among Polish and Romanian community members (CMs) in England, and related access to primary healthcare (PHC) | Polish and Romanian community members (n=30)  [London, Lincolnshire and Berkshire] | Childhood immunisation generally | The Social Ecological Model | Qualitative (semi-structured interviews) | P |
| 1. Guillaume (2004) | To identify the information needs of parents of children under the age of five in relation to MMR vaccination | Parents (n=17)  [Sheffield] | MMR | N/a | Qualitative (semi-structured interviews) | P |
| 1. Ellis (2020) | To explore the interaction between G&T mothers of children aged 0–10 years old, health professionals and their communities and how this impacts upon their decision-making around childhood immunisations | GRT mothers of 0–10-year-old children (n=7)  [Kingston, south-west London] | Childhood immunisation generally | N/a | Qualitative (group AND individual semi-structured interviews) | P |
| 1. Smailbegovic (2003) | To explore the knowledge, attitudes and concerns with respect to immunization and vaccine-preventable infections in parents whose children have not completed the recommended course of immunization | Parents with 1+ immunisation defaults (n=68, questionnaires; n=10 interviews)  [London Borough of Hackney] | Childhood immunisation generally | N/a | Mixed methods ([1] questionnaires, [2] semi-structured interviews: sub-sample) | P |
| 1. Lewendon (2002) | To explore the reasons why some children were not being immunised with the aim of identifying areas of improvement locally in order to increase uptake | Parents (n=44 questionnaires, n=16 focus groups)  [South Devon - a rural, relatively affluent area] | Childhood immunisation generally | N/a | Mixed methods (questionnaires AND focus groups) | P |
| 1. Jackson (2016; 2017) | Aims  1. Investigate barriers to and facilitators of acceptability and uptake of immunisations among six Traveller communities across four UK cities  2. Identify possible interventions to increase uptake of immunisations in these Traveller communities that could be tested in a subsequent feasibility study  Objectives  1. Investigate the views of Travellers on the barriers to and facilitators of acceptability and uptake of immunisations and explore their ideas for improving immunisation uptake  2. Investigate the views of service providers on the barriers to and facilitators of uptake of immunisations within the Traveller communities with whom they work, and explore their ideas for improving immunisation uptake  3. Examine whether or not and how these responses by Travellers and service providers vary within and across communities and for different vaccines (childhood and adult)  4. Use the data collected from 1–3 to identify possible interventions to increase uptake of immunisations in the six Traveller communities  5. Conduct workshops in each community to discuss findings and to produce a prioritised list of potentially feasible and acceptable interventions to be considered for testing in a subsequent feasibility study | Travellers from six communities – Romanian/Slovakian Roma, English Gypsy, Irish Traveller and Scottish Showpeople (n=174, interviews; n=51 workshop)  [Bristol, York, Glasgow, and London] | Childhood immunisation generally | The Social Ecological Model | Qualitative ([1] semi-structured interviews, [2] workshop: sub-sample) | P |
| 1. Johnson (2014) | To explore the ways in which, in the focus group, mothers make sense of, and work with, varying advice and information (both from professional and non-professional sources), within their specific contexts and circumstances, particularly in relation to the MMR and vaccinations, and identify how this is mediated by positionings, practices and relationships | Mothers of preschool children (n=5)  [North of England] | MMR | N/a | Qualitative (focus groups) | P |
| 1. Letley (2018) | To understand the barriers and enablers to vaccination | Charedi Orthodox Jewish community (n=126, survey, n=10, interview) [London borough of Hackney] | Childhood immunisation generally | The WHO Tailoring Immunization Programmes (TIP) approach | Mixed methods ([1] questionnaires, [2] semi-structured interviews: sub-sample) | P |
| 1. Smith (2017) | This paper presents data from a series of focus groups with GRT mothers on their decisions with regard to MMR immunisation and the factors underpinning those decisions. The findings are positioned within a critical realist framework, thus allowing for an exploration of the relative roles of cultural and structural factors in shaping health-related behaviour. Of particular relevance is the proposition that social phenomena are the result of various interacting causal mechanisms: events that occur due to necessity (or the functioning of causal mechanisms) are distinguished from contingent events (occurring due to factors unrelated to those mechanisms) (Dunn, 2012). Disentangling mechanisms that result from deeper social processes and are persistent, cumulative and have tangible outcomes in the social patterning of MMR coverage, from factors that are either extraneous or only tangentially related to the phenomenon in question is the aim of this article. | Site-dwelling GRT women (n=16)  [Kent, South East England] | MMR | N/a | Qualitative (focus groups) | P |
| 1. McHale (2016) | To identify factors that continue to affect MMR uptake rates, and con- sider key issues related to parents or carers of confirmed measles cases. | Parents or carers of unvaccinated measles cases (n=47)  [Merseyside] | MMR | N/a | Qualitative (semi-structured interviews) | P |
| 1. Henderson (2008) | To investigate whether there are specific religious or ethnic reasons for low uptake of immunization amongst orthodox Jewish families in North East London and explores perceptions of barriers such as larger family size, the role of local health care services, and the significance of wider sources such as local and national media reporting. | Orthodox Jewish mothers (n=25) [Hackney] | Childhood immunisation generally | N/a | Qualitative (semi-structured interviews) | P |
| 1. Tickner (2010) | To identify possible reasons for lower uptake of pre-school immunizations, compared with the primary course. | Parents (n=21)  [Southampton, Romsey, Windsor] | MMR 2 AND dTaP/IPV booster | N/a | Qualitative (semi-structured interviews) | P |
| 1. Bell (2020) | To explore factors influencing vaccination behaviours amongst Romanian and Roma Romanian communities in these three cities. | Romanian and Roma Romanian parents (n=9)  [Birmingham, Leeds and Liverpool] | Childhood immunisation generally | 5As Taxonomy for Determinants of Vaccine Uptake (Access, Affordability, Awareness, Acceptance and Activation) | Qualitative (semi-structured interviews) | P |
| 1. Lunts (2002) | To determine parent's reasons for non-uptake of MMR vaccine in the inner city since the adverse publicity allegedly linking this vaccine to autism and bowel disease and to determine how well these reasons were understood by their health visitors and GPs. | Parents who had not received their first MMR vaccine since the adverse publicity (n=93) [Bristol] | MMR | N/a | Mixed Methods (questionnaire) | P |
| 1. Bennett (1992) | We are aware of no research which has attempted to measure differences on such measures between parents who do and do not vaccinate their child. The present study sought to investigate such differences. | Parents (n=228) [Mid Glamorgan, Wales] | Childhood immunisation generally | The Health Belief Model | Quantitative (structured interview) | P |
| 1. Sampson (2011) | To explore parental reasons for non-uptake of influenza vaccination in young at-risk groups. The study hypothesis was that exploration of parental reasons for non-uptake may reveal important barriers to an effective influenza vaccination programme. | Parents of children identified as being in at-risk group for influenza vaccination but who had not received vaccination (n=16, questionnaire; n=7, questionnaire) [Inverness, Scotland] | Influenza | N/a | Qualitative ([1] questionnaire, [2] questionnaire which could be done in written/ interview format: sub-sample) | P |
| 1. Lakhani (1987) | A three stage investigation of possible factors contributing to the low uptake of measles immunisation was carried out. These stages are (a) a study of the SETRHA versions of the standard child health computer systems (its functioning and the transmission of information between the various service providers and the computer centre) to establish whether the low uptake of measles immunisation could be partly due to a malfunction in the total information network; (b) a study of the attitudes and beliefs of parents about measles immunisation and parental socioeconomic factors that are associates with the uptake of service in Maidstone; and (c) a study of the knowledge, attitudes, and beliefs of health professionals about measles immunisation and their potential influence on the uptake of the service. | Parents (n=174) [Maidstone] | Measles | N/a | Unknown (interviews – type unspecified) | P |
| 1. Condon (2002) | To explore the attitudes of ethnic minority parents to preschool immunisations, particularly first MMR (measles, mumps and rubella vaccination). | Mothers of children of Pakistani, Somali and Afro-Caribbean ethnicity (focus groups n=14, interviews n=7) [Bristol] | MMR1 | N/a | Qualitative (focus group AND interviews) | P |
| 1. McMurray (2004) | To explore parents’ accounts of decision making relating to the MMR vaccine controversy, identifying uptake determinants and education needs. | Parents (n=69) [Leeds] | MMR | N/a | Qualitative (semi-structured interviews) | P |
| 1. Morgan (1987) | Since routinely offered immunisation is well accepted the poor levels of protection amongst first generation immigrants suggests lack of knowledge concerning infectious diseases and the details of protection. This study investigated such a possibility. | Asian and white parents (n=64) [Nottingham] | Childhood immunisation generally | N/a | Quantitative (structured questionnaire) | F (but results only ¾ of a page). |
| 1. Bedford (2006) | As part of a survey of parents' views about the acceptability of potential vaccines we also gathered views about information relating to immunisation. We investigated the sources of information used by parents, which sources help them to decide whether or not to accept vaccines, parents' views on information given and their preferences for the timing of receipt of information about immunisation. | Parents (n=859) [East Berkshire] | Childhood immunisation generally | N/a | Mixed methods (questionnaire) | P |
| 1. Hill (2013) | The principal aim of the feasibility study described in this article is to ascertain influencing factors on parental immunisation decision making. This study seeks to: Explore the factors that influence parental decision making  on whether to immunise their child with the MMR vaccine; Ascertain from whom the parent seeks information in their immunisation decision making on the MMR vaccine. | Parents of children who had received the vaccine (n=5) [London] | MMR | N/a | Qualitative (semi-structured interviews) | P |
| 1. Austin (2001) | To explore parents’ experiences of deciding to have their child immunised, with a view to making changes in practice, if indicated, in: parents’ knowledge of the immunisation programme; communication between parents and professionals; communication between members of the PHCT; consistency of information provided to parents by the PHCT. | Parents (n=13) [Unspecified] | Childhood immunisation generally | N/a | Qualitative (semi-structured interviews) | P |
| 1. Cuninghame (1994) | To measure immunization uptake and to identify reasons for non- uptake, attitudes towards immunization and immunization services, and to identify areas for improvement. | Orthodox Jewish parents (n=67) | Childhood immunisation generally | N/a | Mixed methods (questionnaire: could be completed in person, via telephone, or post) | P |
| 1. Adjaye (1981) | Some social and secular factors that have affected measles vaccine uptake in two communities in South London were identified in a survey of families with children eligible for vaccination. | Parents (n=258) [South London] | Measles | N/a | Mixed methods (questionnaire: conducted in-person) | P |
| 1. Newton (2006) | To explore their views on these issues, we con- ducted a postal survey of parents in July 2002 to explore the acceptability of this vaccine being added to the UK immunisation schedule and to evaluate parents’ knowledge of pneumococcal disease. | Parents (n=601) [Oxfordshire] | PCV | N/a | Quantitative (questionnaire) | P |
| 1. Gorman (2019) | To explore Polish migrant women’s views on the childhood vaccination programme in Edinburgh, Scotland, in the context of the trust held in various aspects of the programme and with a specific focus on influenza and HPV vaccination. | Female Polish migrants [Lothian, Edinburgh, Scotland] | Influenza | N/a | Qualitative (focus groups) | P |
| 1. Morgan (1987b) | To examine a number of possible explanations for failure to take up measles immunization : (1) parents' dissatisfaction with or problems of access to child health services; (2) parents' general attitudes to and knowledge about measles immunization and their perception of the seriousness of the disease; (3) the role of doctors, health visitors, and relatives and friends in encouraging or discouraging immunization and; (4) specific problems which result in delay or non-uptake. | Parents of children with missed measles immunisation (n=20) [Maidstone, Kent] | Measles | N/a | Unknown (interviews) | P |
| 1. Bell (2021) | To explore parents’ and guardians’ views and experiences of accessing National Health Service (NHS) general practices for routine childhood vaccinations during the coronavirus (COVID-19) pandemic in England. | Parents (n=1252 questionnaire, n=19 interview) | Childhood immunisation generally | COM-B | Mixed methods ([1] questionnaire, [2] semi-structured interviews: sub-sample) | P |
| 1. New (1991) | This paper presents qualitative data to emerge from a largely quantitative case-control study of the uptake of infant immunisation in two District Health Authorities in the North West of England. It was hypothesised that problems arising from transport and time-space constraints would distinguish the parents of those children who had missed appointments from those who had attended. | Parents (n=253) [Lancaster and Salford] | Childhood immunisation generally (a primary course of three injections to protect against diphtheria, tetanus, per- tussis and polio) | N/a | Mixed methods (questionnaire) | P |
| 1. Yarwood (2005) | The main objectives of the research were to:  • obtain information on mothers’ knowledge of immunisation;  • obtain information on mothers’ attitudes towards immunisation;  • obtain information on mothers’ experience of immunisation services;  • monitor the recall and interpretation of NHS Immunisation Information (NHS II) advertising and immunisation information materials. | Parents (n=1000) [Unspecified] | Childhood immunisation generally | N/a | Quantitative (questionnaire) | P |

*Note, this table reports methods based on data extracted. For instance, a study may be in face mixed methods, but will be reported here as qualitative if the quantitative component of the study did not meet the inclusion criteria (e.g., only used routine uptake data as a measure of accessibility).*

**Intervention studies**

| **Author (year)** | **Intervention type** | **Intervention [development]** | **Participants (n) [setting]** | **Vaccines considered** | **Guiding theory/ model** | **Method (study design)** | **Results** |
| --- | --- | --- | --- | --- | --- | --- | --- |
| 1. Lakhani (1984) | Communication/ information | A home based record to keep note of child health, including immunisations. Also had an accompanying health education booklet.  [Developed by a ‘multidisciplinary group’]. | Parents (n=322) [West Lambeth] | Childhood immunisation generally | N/a | Mixed methods (quantitative [randomised controlled trial] and qualitative [interviews and postal questionnaires]). | No significant difference. Information being recorded on a previously issued immunisation card. In fact, 52% of booklets has this card inside the front flap. This immunisation card was since discontinued and edits were made to the booklet. Benefits were reported in terms of improving communication. |
| 1. Atchison (2013) | Communication/ information | To improve uptake of childhood immunizations in Wandsworth we developed a standardized call/recall system based on parents being sent three reminders and defaulters being referred to a Health Visitor.  [Designed based on national evidence-based guidance and local/regional good practice approaches]. | Thirty-two practices [Wandsworth] | Childhood immunisation generally | N/a | Mixed methods (qualitative [structured interviews] and quantitative [comparison or pre- and post-intervention uptake rates]). | The call/recall system was viewed positively by both parents and staff. Most children due or overdue immunizations were successfully captured by the 1st invitation reminder. After three invitations, between 87.3 % (MMR1) and 92.2 % (pre-school booster) of children identified as due or overdue immunizations successfully responded. Prior to implementation there was no difference in uptake rates between intervention and non-intervention practices. Post- implementation uptake rates for DTaP/IPV/Hib, MMR1, MMR2 and the pre-school booster were significantly greater in the intervention practices. Similar findings were seen for PCV and Hib/MenC boosters, although the differences were not statistically significant at the 5 % level. |
| 1. Carter (2005) | Communication/ information | A coordinated health education programme aimed at doctors, health visitors, and parents and included the publication and distribution of local measles immunisation guidelines, a monthly community health report covering all aspects of communicable health diseases and immunisation, and regular press, radio, and television reports and appearances. All these efforts were regularly reinforced by meetings and discussions with the staff concerned.  [Strategy devised at staff meeting.] | Parents (n=91) and HCWs (n=99)*  [Fife]  *Note this is not related to the general vaccine uptake data. | Measles | N/a | Quantitative (comparing trends in immunisation rates pre- and post-intervention). | An increase of 13% in vaccine uptake in the area compared to 7% increase throughout Scotland during the same period. |
| 1. Lwembe (2016) | Communication/ information | This study was undertaken to assess the suitability, feasibility and acceptability of the Celebrate and Protect programme across nine boroughs in London.  [Iterative development with stakeholders, including tailored rollout.] | Parents (n=31) and HCWs/policy leads/ stakeholders (n=24)  [London] | Childhood immunisation generally | 1. Unnamed conceptual model (for intervention)  2. Johnson and Sholes (2005) suitability, feasibility and acceptability framework (for evaluation) | Qualitative (semi-structured telephone interviews and focus groups). | The Celebrate and Protect programme was seen as an innovative collaborative programme to engage with parents and carers of children in order to improve relationships between service users and providers and subsequently increase vaccination uptake. The analysis demonstrates that that the celebration card is suitable for its purpose, acceptable to both healthcare professionals and to parents/carers of children and the Celebrate and Protect programme has been able to deliver its aims. Whilst the delivery of the ‘celebration card’ intervention in primary met its objectives there are some outstanding issues in terms of the sustainability of the initiative and the ability to demonstrate quantitative improvements in vaccination uptake rates. |
| 1. Conway (1999) | Location  Financial  Organisation  Communication/ information | Immunisation status according to the child’s principal carer was checked against official records. Junior doctors were instructed to offer appropriate vaccination before discharge, and consultants were asked to reinforce this proactive policy on ward rounds.  [Not specified] | Carers of preschool age children admitted to a paediatric ward (n=1000) [York] | Childhood immunisation generally | N/a | Quantitative (uptake data). | Excluding those children who were not fully immunised against pertussis through parental choice, 142 children (14.2%) had missed an age appropriate immunisation and 41 were due a scheduled immunisation. None had a valid contraindication. Only 43 children were offered vaccination on the ward but up-take was 65% in this group. Admission to hospital provides opportunities for catch up and routine immunisations and can contribute to the health care of an often disadvantaged group of children. These opportunities are frequently missed. Junior doctors must be encouraged to see opportunistic immunisation as an important part of their routine work. |
| 1. Jackson (2011) | Communication and information | A parent-centred, multi-component intervention (balanced information, group discussion, coaching exercise) on informed parental decision-making for MMR.  [Informed by a systematic review and an interview study with parents.] | Parents of children eligible for MMR vaccination (n=66) | MMR | It is also in line with the fundamental tenets of health promotion that is based on an ‘engagement’ model of communication where a key goal is empowerment. | Quantitative (cluster randomised controlled trial). | Significantly more parents in the intervention arm reported vaccinating their child (93% versus 73%, p = 0.04). |
| 1. SQW (2011) | Location  Supply/demand  Financial  Organisation  Communication/ information | The Flying Start programme was launched by the Welsh Government and aimed ‘to make a decisive difference to the life chances of children aged under four in the areas in which it runs’. It is administered as a grant to local authorities to provide intensive assistance to children and their families within specific catchment areas (varied intervention in the form of outreach, parenting programmes, childcare, etc.). As part of Flying Start’s aims of improving child health, the programme encourages parents to vaccinate their children against a number of preventable diseases and illnesses including measles, polio and diphtheria, among others.  [Not specified] | Parents (n=3211) [Wales] | Childhood immunisation generally | Self-generated logic framework | Quantitative (quasi-experimental survey design). | No impact from Flying Start is observable from the impact analysis on take-up of immunisations. |

*Note, only intervention elements/results which met the inclusion criteria are reported here – namely, intention to vaccinate is not reported.*

*Jackson (2011) reports both intentions to vaccinate and actual vaccine uptake – only actual vaccine uptake is reported here.*

*SQW (2011) reports the outcome of a generalised intervention on a number of health domains, only immunisation as a parental behaviour outcome is reported.*
